# *Wolbachia* Infections Mimic Cryptic Speciation in Two Parasitic Butterfly Species, *Phengaris teleius* and *P. nausithous* (Lepidoptera: Lycaenidae)

**DOI:** 10.1371/journal.pone.0078107

**Published:** 2013-11-06

**Authors:** Sylvia Ritter, Stefan G. Michalski, Josef Settele, Martin Wiemers, Zdenek F. Fric, Marcin Sielezniew, Martina Šašić, Yves Rozier, Walter Durka

**Affiliations:** 1 Helmholtz Centre for Environmental Research, Department of Community Ecology, Halle (Saale), Germany; 2 German Centre for Integrative Biodiversity Research (iDiv) Halle-Jena-Leipzig, Leipzig, Germany; 3 Biology Centre, Academy of Sciences of the Czech Republic, České Budějovice, Czech Republic; 4 University of Bialystok, Institute of Biology, Department of Invertebrate Zoology, Białystok, Poland; 5 Croatian Natural History Museum, Department of Zoology, Zagreb, Croatia; 6 CED Entreprises, Centre d’activités de Gorge de Loup, Lyon, France; Onderstepoort Veterinary Institute, South Africa

## Abstract

Deep mitochondrial divergence within species may result from cryptic speciation, from phylogeographic isolation or from endosymbiotic bacteria like *Wolbachia* that manipulate host reproduction. *Phengaris* butterflies are social parasites that spend most of their life in close relationship with ants. Previously, cryptic speciation has been hypothesised for two *Phengaris* species based on divergent mtDNA sequences. Since *Phengaris* species are highly endangered, the existence of cryptic species would have drastic consequences for conservation and management. We tested for cryptic speciation and alternative scenarios in *P. teleius* and *P. nausithous* based on a comprehensive sample across their Palaearctic ranges using COI gene sequences, nuclear microsatellites and tests for *Wolbachia*. In both species a deep mitochondrial split occurring 0.65–1.97 myrs ago was observed that did not correspond with microsatellite data but was concordant with *Wolbachia* infection. Haplotypes previously attributed to cryptic species were part of the *Wolbachia*-infected clades. In both species remaining phylogeographic structure was largely consistent between mitochondrial and nuclear genomes. In *P. teleius* several mitochondrial and nuclear groups were observed in East Asia while a single haplogroup and nuclear cluster prevailed across continental Eurasia. Neutrality tests suggested rapid demographic expansion into that area. In contrast, *P. nausithous* had several mitochondrial and nuclear groups in Europe, suggesting a complex phylogeographic history in the western part of the species range. We conclude that deep intraspecific divergences found in DNA barcode studies do not necessarily need to represent cryptic speciation but instead can be due to both infection by *Wolbachia* and phylogeographic structure.

## Introduction

Cryptic species, i.e. the presence of phylogenetically distinct units within a morphologically defined taxon [1], are a common phenomenon among all animal taxa and biogeographical regions. They can seriously confuse taxonomy based solely on morphological characters [2]. More importantly, cryptic speciation affects our understanding of biodiversity and its conservation [1]. Arthropods are expected to contain many cryptic species and in particular parasitic species seem to have a higher evolutionary potential than free-living species and are potential candidates for cryptic speciation [3]. DNA barcoding using the mitochondrial gene Cytochrome *c* Oxidase I (COI) has become a standard method to assign unknown individuals to species, to assess biodiversity, and to discover new species including cryptic units within well-defined morphospecies [4], [5]. For some of the cryptic units it has additionally been shown that they correspond well with a divergent ecological niche [6].

However, the sole use of mtDNA sequences as a tool for species detection and delimitation can be problematic [7], [8]. Patterns of deep divergence of mitochondrial DNA sequences within species may be due to historical processes like introgression between species [9], or phylogeographic isolation [10]. Furthermore, in invertebrates the assumption of neutral evolution of mtDNA may not be met due to the presence of endosymbiotic bacteria [11], [12], [13]. The common microbial endoparasite *Wolbachia* often manipulates the reproductive system of its host thus enhancing its own transmission to the next generation [11], [14]. When a *Wolbachia* infection has no detrimental fitness effects on the host [15], [16] and the host has no *Wolbachia* suppressing elements [17], the infection can spread through whole populations and species to fixation [17], [18]. As a consequence of the maternal inheritance of the infection this may lead to a selective sweep and fixation of the mitochondrial haplotype of infected individuals [15]. However, a number of empirical studies have found that selfish genetic elements like *Wolbachia* are maintained within populations at relatively low frequencies [17], [19]. Under which conditions *Wolbachia* persists at low frequency, thus maintaining mitochondrial polymorphism, is less clear as fixation frequency depends on various factors like reproductive fitness effects, population size and structure, infection and transmission frequency, bacterial density and/or phage presence [15], [20], [21], [22]. Furthermore, fitness effects of *Wolbachia* on host individuals can be conditional on environmental factors [23], [24]. An important consequence of *Wolbachia* infection is its influence on mtDNA patterns which may seriously undermine the power of DNA barcoding for species detection. It can either mask species diversity due to mtDNA introgression between species [18], [25]. Or, in contrast, it can promote high mtDNA divergence due to long lasting reproductive isolation between infected and uninfected lineages and may even lead to the formation of new species [26]. *Wolbachia* may also become lost because of inefficient transmission [27] which may further complicate the interpretation. Hence, to assess whether an observed mtDNA haplotype pattern was the result of *Wolbachia* infection, additional analyses are needed including tests for the presence of *Wolbachia* and the use of additional nuclear markers [9], [28], [29].

Species with a parasitic lifestyle are both potential candidates for cryptic speciation [3] and particularly prone to be horizontally infected by endoparasites like *Wolbachia* [30]. Butterflies of the genus *Phengaris* Doherty, 1891 (formerly *Maculinea* van Eecke, 1915; Lepidoptera: Lycaenidae) exhibit a parasitic phase within their life cycle [31]. In their last larval stage caterpillars show numerous evolutionary adaptations to an intricate nest parasitism of *Myrmica* ant species [32]. *Phengaris* are rare and threatened species listed in the European Habitats Directive and of high importance for nature conservation [33]. Thus, cryptic biodiversity could potentially have impacts on the evaluation of their vulnerability and conservational status and on management strategies. In Europe, populations have suffered from local extinctions for decades, mainly because of changes in local farming practices of grasslands, the main habitat of the species [34]. Evidence for a high infection rate with endoparasites exists for two *Phengaris* species. In populations of *P. alcon* from Poland and Lithuania as well as *P. arion* from Poland and Italy all screened samples turned out to be infected with *Wolbachia* strains of supergroup B and A, respectively [35], [36]. Out of the total of 11 *Wolbachia* supergroups these two are currently the only ones known to occur in butterflies, where supergroup B is prevalent [37]. In the genus *Phengaris* 12 species are currently recognized [38], [39]. The two co-occurring, closely related species studied in detail here, *Phengaris teleius* (Bergsträsser, [1779]) and *Phengaris nausithous* (Bergsträsser, [1779]), share *Sanguisorba officinalis* (Rosaceae) as their only foodplant. *P. teleius* is morphologically variable and a number of subspecies have been described from Asia [40]. In contrast in *P. nausithous* most authors only recognize the nominate form (but see Rákosy et al. [41] who recognize subspecies *kijevensis* Sheljuzhko, 1928 in Eastern Europe). Both species have wide and overlapping distribution areas in temperate regions of the Palaearctic [42]. Phylogenetic hypotheses of the genus based on morphological, ecological [43] as well as molecular evidence [38], [44], [45] have been formulated. Cryptic speciation has explicitly been hypothesized for *P. nausithous* [44], *P. teleius* [45] or both [38] based on a limited number of divergent sequences. Alternatively, however, high sequence variation and the large morphological variability of *P. teleius* could be a result of its phylogeographic history, e.g. by isolation in different pleistocene refugia, or may involve endosymbiotic bacteria. However, a comprehensive phylogenetic and phylogeographic analysis that also considers the potential contribution of *Wolbachia* infection is still lacking.

Here we present a phylogenetic analysis of *Phengaris teleius* and *P. nausithous* based on a comprehensive sample across their Palaearctic ranges. We use COI sequences of mitochondrial DNA as well as nuclear microsatellite markers to test for cryptic speciation. Furthermore, we test whether distinct lineages can be explained by an association with *Wolbachia* infections. Finally, we use the data to analyse phylogeographic patterns of *P. teleius* and *P. nausithous*. Our study thus provides further insights into postglacial movement patterns of Palaearctic insect species.

## Materials and Methods

### Sampling

**Figure 1:**
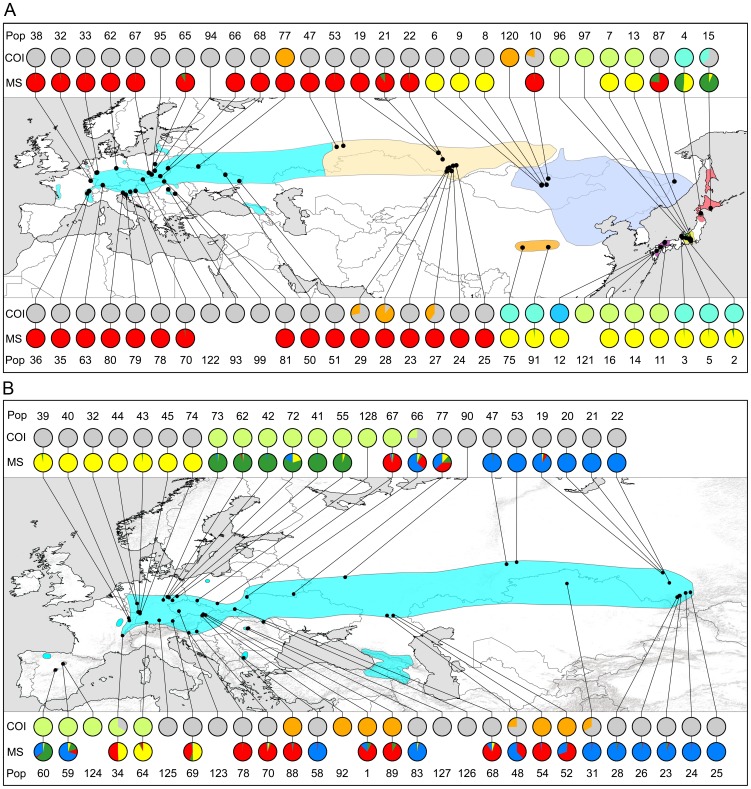
Distribution range, sampling sites and genetic structure at mitochondrial and nuclear genomes. COI = cytochrome oxidase I (for details see Figure 2) and MS = microsatellites (for details see Figure 4) for *Phengaris teleius* (**A**) and *P. nausithous* (**B**). For details on populations, see Table S1. Distribution ranges are based on records (Europe: [42], [105]; Asia: [106], [107], [108], [109] and expert knowledge (Asia: personal communication Kosterin, Wang). Shading color corresponds to subspecific affiliation of *P. teleius* according to Table S1 (cyan: nominate species, peach: *P. t. obscurata*, blue: *P. t. euphemia*, orange: *P. t. sinalcon*, pink: *P. t. ogumae*, yellow: *P. t. kazamoto*, green: *P. t. hosonoi*, violet: *P. t. daisensis*).

*Phengaris teleius* (Bergsträsser, [1779]) including the subspecies *sinalcon* Murayama, 1992 [46], *obscurata* Staudinger, 1892 [47], *euphemia* Staudinger, 1887 [48], *hosonoi* Takahasi, 1973 [49], *kazamoto* Druce, 1875 [50], *ogumae* Matsumura, 1910 [51] and *daisensis* Matsumura, 1926 [52] and *Phengaris nausithous* (Bergsträsser, [1779]) were sampled throughout their distribution ranges from 44 and 36 populations, respectively (Fig. 1, Table S1). Note that *P. teleius* and *P. nausithous* co-occurred in 19 populations. Up to 10 individuals were sampled per species and location. Hand netted adults (N = 110) were killed with potassium cyanide and kept either in glassine envelopes or in 99.8% ethanol. Caterpillars (N = 149) taken from the food plant *Sanguisorba officinalis* were conserved in ethanol. We could not assess sex ratio as sample sizes per population were too low and more than half of the specimens were larvae, for which sex could not be assessed. Collection permits were obtained from Struktur- und Genehmigungsdirektion Nord (Koblenz, Germany), Regierungspräsidium Leipzig (Germany), Regierung von Unterfranken (Würzburg, Germany), Thüringer Landesverwaltungsamt (Weimar, Germany), and specimen collectors’ own collection permits, if required in respective countries.

### DNA Barcoding and Tests for *Wolbachia* Infection

Total genomic DNA was extracted using the QIAGEN DNeasy Blood & Tissue Kit (QIAGEN, Hilden, Germany), following the manufacturer’s instructions. Two fragments of the COI gene were amplified using the primer combinations: LCO – Nancy and Tonya – Hobbes [45]. PCR was performed in 20 µl reactions, containing 0.5 pmol of each primer, 200 µM dNTPs, PCR buffer, 1.875 mM MgCl_2_, and 0.8 units Fermentas Taq DNA polymerase (Fermentas, Leon-Rot, Germany). The thermocycler protocol was: denaturation at 95°C (2 min) followed by 37 cycles of 95°C (1 min), 47°C (1 min) and 72°C (1.5 min), and a subsequent final extension step at 72°C (10 min). PCR-products were directly cycle-sequenced using the ABI BigDye Terminater v3.1 cycle sequencing Kit using the same primers. Products were sequenced on an Applied Biosystems 3130×l Genetic Analyzer (Applied Biosystems, Foster City, USA). About 10% of the fragments were treated with a multiple tube approach and 20% of the fragments were sequenced in both directions which did not show any mismatches. Sequences were obtained for 147 samples of *P. teleius* and 112 samples of *P. nausithous*. GenBank accession numbers for all concatenated sequences are provided in Table S1.

We tested all individuals for infection with *Wolbachia* performing two independent PCR screens for the *Wolbachia* surface protein (*wsp*) following Zhou et al. [53]. PCR products were visualized on 1.5% agarose gels and scored for the presence of *Wolbachia* infections (Table S1). The *wsp*-genes of *Wolbachia* endosymbionts of 8 *P. teleius* and 3 *P. nausithous* were sequenced to determine allele and supergroup correspondence, using the Wolbachia *wsp* Database [54] and BLAST.

### mtDNA Sequence Analysis

COI fragments of 147 and 112 individuals of *P. teleius* and *P. nausithous*, respectively, were manually concatenated and aligned with BioEdit [55]. To avoid the inclusion of mitochondrial pseudogenes [56], translated amino acid sequences were tested for substitutions and stop-codons using the program MEGA 5 [57]. In both *P. teleius* and *P. nausithous* ten non-synonymous substitutions were found leading to a change in the amino acid sequence. However, we did not regard these substitutions as indicative for a pseudogene because the mutations occurred in parts of the protein known for their high amino acid variability [58], [59] or because substitutions led to amino acids of similar characteristics. Additionally, we added all published COI/COII sequences of *P. teleius* and *P. nausithous* available from GenBank as of 1 June 2013 (Table S1). These also included all publicly available barcode sequences from the barcode of life database (BOLD; [60]). Note that the test for *Wolbachia* infection could not be performed for sequences retrieved from GenBank. As outgroup taxa sequences of further *Phengaris* species (*P. arion* (Linnaeus, 1758), *P. alcon* ([Denis & Schiffermüller], 1775), *P. albida* Leech, 1893, *P. atroguttata* (Oberthür, 1876), *P. daitozana* Wileman, 1908) were taken from GenBank (Table S1).

A haplotype analysis was carried out using TCS 1.21 [61]. Prior to this analysis, parts of our alignment which were only available for a minority of sequences (alignment positions 1–60, 649–766 and 1193–2210) were removed, and sequences were sorted according to the number of non-ambiguous sites in decreasing order. All short sequences (below 680 bp, i.e. all short barcode sequences) which were included in the first haplotype analysis were removed from further analysis due to the low level of overlap resulting in 157 sequences for *P. teleius* and 120 for *P. nausithous*. Gene evolution was visualized with a haplotype network using statistical parsimony as implemented in TCS 1.21 using default options.

Phylogenetic trees were inferred applying two criteria, i.e. unweighted Maximum Parsimony (MP), and Maximum Likelihood (ML), using the consensus haplotype sequences of the complete alignment. MP analysis was conducted in MEGA 5 [57] doing a heuristic search (Close-Neighbor-Interchange algorithm). Initial trees were obtained by random addition of sequences (10 replicates). All codon positions were included and alignment gaps were treated as missing data. For ML inference, the Tamura-Nei model [62] with a gamma distribution for rate variation among sites (G = 0.084) was selected using jModelTest 0.1.1 [63] as the best fitting evolutionary model. Tree searches were performed with PhyML version 3.0 [64] using the SPR search option and a BIONJ starting tree. Branch support for MP- and ML-trees was estimated by bootstrapping the dataset 500 times.

Average sequence divergence for COI was calculated as uncorrected pairwise *p*-distances of all haplotype sequence pairs within and between clades using MEGA 5 [57]. Because fossil data of *Phengaris* are not available, and geological events cannot be linked with branching events in our trees, we calculated age estimates of splitting events by using three COI substitution rates reported for arthropods, i.e. 1.3% [65], 2.3% [66], and 3.5% per million years [67].

We estimated nucleotide diversity π [68]. To test whether sequence diversity was concordant with expectations of neutral evolution we computed Tajima’s *D* [68] and Fu’s *F* [69] as implemented in Arlequin 3.5.1.2 [70]. Deviations from neutral evolution may suggest recent demographic expansions or bottlenecks. For these analyses only nucleotide positions represented in every sample were included (*P. teleius* N = 845; *P. nausithous* N = 768). Furthermore, we excluded samples from divergent “Wolbachia” clades.

### Nuclear Microsatellite Analysis

Samples were genotyped at eight nuclear microsatellite loci (Macu1, Macu3, Macu7, Macu8, Macu9, Macu11, Macu15, Macu16; [71]). Loci were amplified in three reactions with a multiplex PCR kit (QIAGEN) using fluorescent labelled primers and separated on an Applied Biosystems 3130×l Genetic Analyzer (Applied Biosystems, Foster City, USA). Individuals for which fewer than four loci yielded interpretable results were excluded from the analysis resulting in a data set of 143 *P. teleius* and 109 *P. nausithous* genotypes.

We used a Bayesian clustering method to assess population structure of individual multilocus genotypes separately for each species using STRUCTURE 2.3 [72]. For each *K* ranging from 1 to 10, we performed 10 replicate runs with 100.000 steps after a burn-in period of 50.000 steps. We used the admixture model without prior population information and with correlated allele frequencies. Most likely *K* values were estimated following Evanno et al. [73]; see Fig. S1. The program CLUMPP 1.1.1 [74] was used to estimate the mean cluster assignment across replicate runs. For the resulting main clusters we calculated gene diversity (*H*_e_), allelic richness (*A*_r_), private allelic richness (*pA*_r_), and shared allelic richness (*sA*_r_) using ADZE 1.0 [75]. Differentiation among clusters was quantified as θ, an estimator of Wright’s *F*_ST_ [76] and as standardized *G’_ST_* ([77], eq. 4b) calculated in Fstat 2.9.3.2. [78]. Individuals with highly ambiguous cluster membership (inferred ancestry <0.8; *P. teleius*: N = 4; *P. nausithous*: N = 24) were excluded from this analysis.

We assessed the relationship between nuclear and mitochondrial genomes by correlating inter-individual genetic distances and testing the significance by a Mantel test with 1000 randomizations in R version 2.12.2 [79]. For microsatellites, genetic distances were quantified as proportion of shared alleles calculated with MSA v. 3.0 [80]. For COI sequences we used Maximum Composite Likelihood estimates with pairwise deletion of missing data and gamma distributed substitution rates among sites, calculated in MEGA 5.

## Results

### *Wolbachia* Infection

In *P. teleius* we found 19 out of 147 (13%) individuals investigated to be infected with *Wolbachia*, while in *P. nausithous* we found 6 out of 112 (5.4%) (Table S1). The *Wolbachia wsp* genes had one allele each in *P. teleius* and *P. nausitous* (GenBank-accession no. JX470438, JX470439). The sequence from *P. teleius* is identical with allele 431 found in Heteroptera from Japan [81]. The sequence from *P. nausithous* differs only slightly from three known alleles (264, 266, 436) detected in Lepidoptera and Hemiptera also originating from Japan [81], [82] and was submitted as new allele 639 to the *Wolbachia wsp* database. The two *wsp* alleles are very distinct (nucleotide *p*-distance: 9.4%; protein *p*-distance: 14%), however, both are affiliated with *Wolbachia* supergroup B.

### Phylogenetic Inference in *Phengaris Teleius* and *P. nausithous*

The final COI+COII alignment contained 282 sequences (157 *P. teleius*, 120 *P. nausithous*, 5 outgroup) with a total length of 2253 bases of which 333 (14.8%) sites were variable and 210 were parsimony informative (9.3%). No indels were detected. In total 124 unique haplotypes were observed (Table S1), 72 in *P. teleius* and 52 in *P. nausithous*. Of these, 3 haplotypes (N50, N51, N52) were observed exclusively in barcode sequences, which were excluded from further analysis. However, these haplotypes only differed in single nucleotide positions from other haplotypes (N06, N42, and N49, respectively).

**Figure 2:**
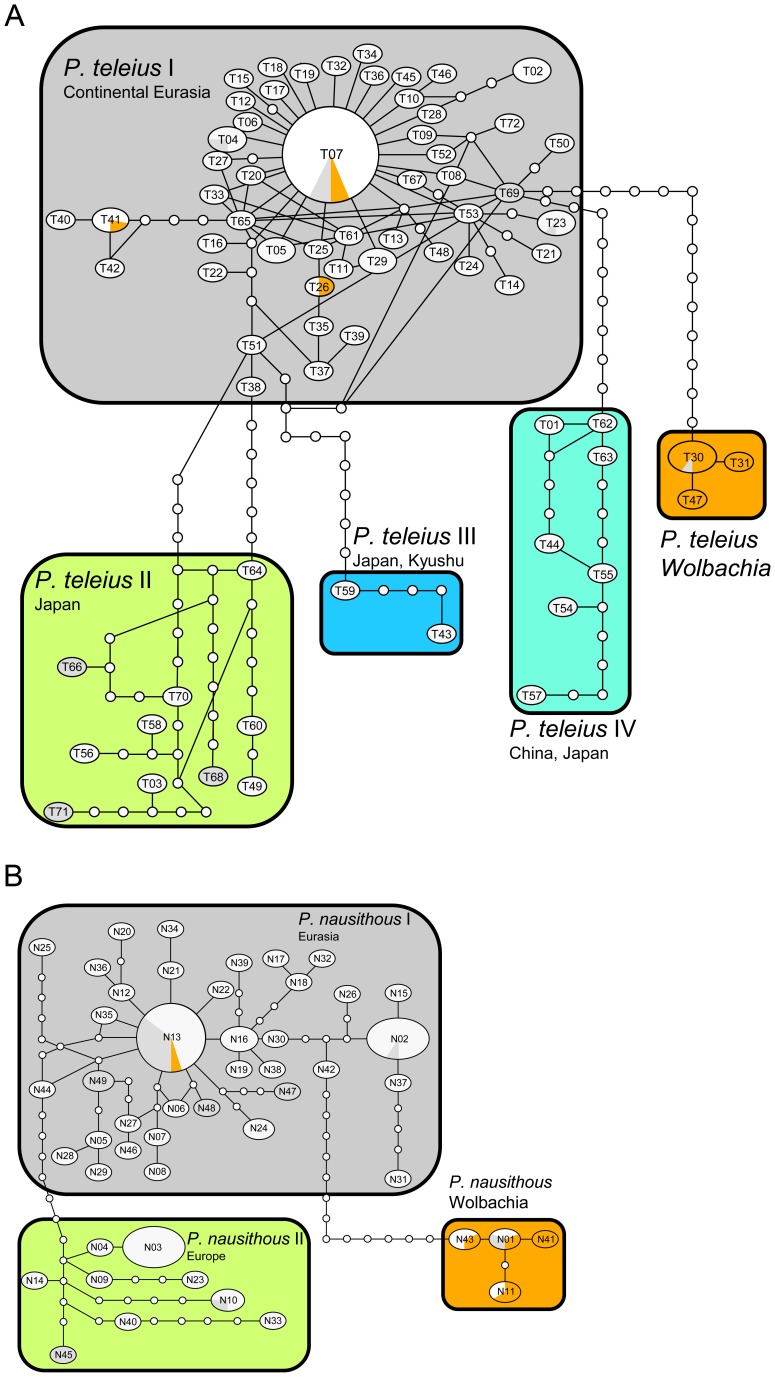
COI haplotype networks for *Phengaris teleius* (A) and *P. nausithous* (B). Circle size is proportional to haplotypes frequency (Table S1). The proportion of individuals infected with *Wolbachia* is indicated by a colored pie chart. Note that in several haplotypes samples could not be tested for *Wolbachia* since the corresponding sequence was extracted from Genbank (grey shading).

**Figure 3:**
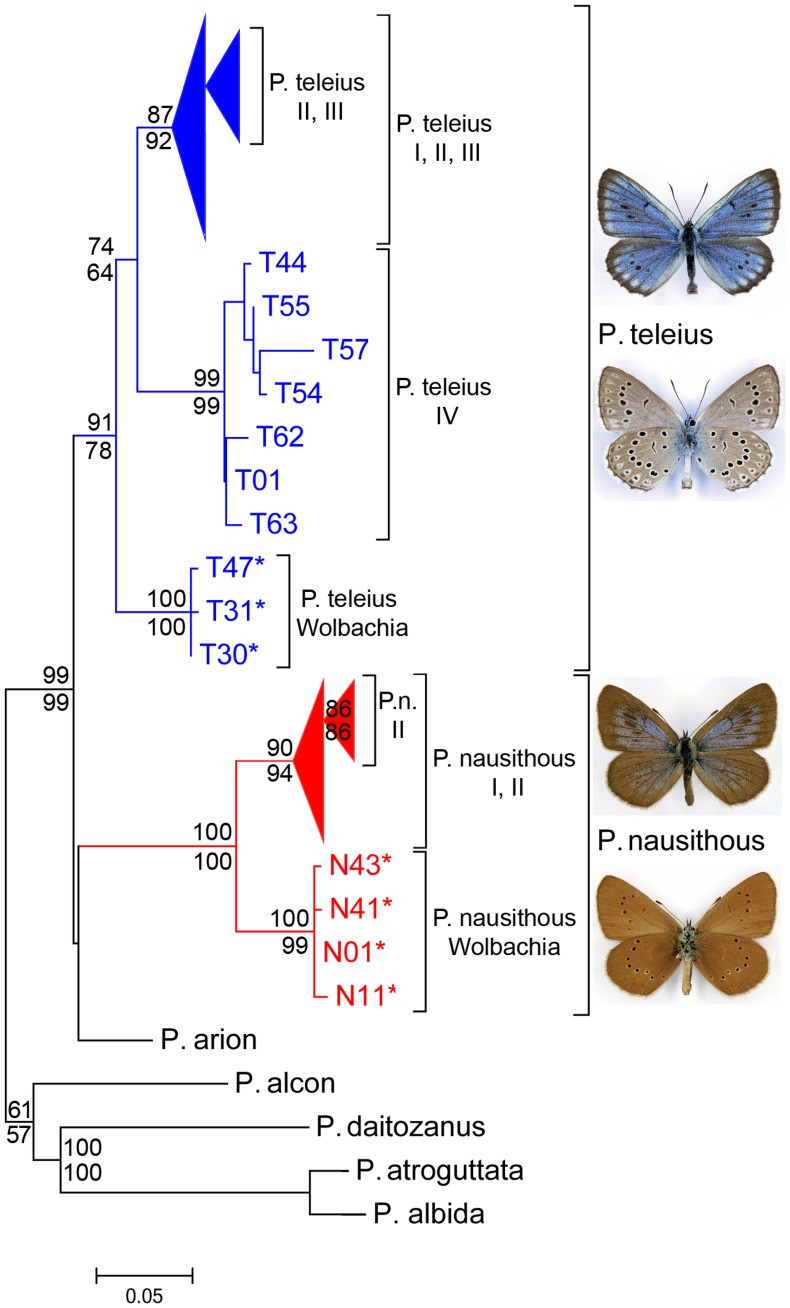
Phylogram for haplotypes of *Phengaris teleius* and *P. nausithous* based on ML analysis for mitochondrial COI. Bootstrap values in percent (>50%) are given above branches (based on ML analysis) and below branches (MP analysis). Bootstrap values within subclades are not shown (see Figure S2, S3). *haplotypes associated with *Wolbachia.*

The COI haplotype network calculation resulted in independent networks for each outgroup species, *P. teleius*, *P. nausithous*, and within both study species a clade dominated by *Wolbachia*-infected individuals under the 95% parsimony limit (0.956 = 13 steps). A slightly relaxed parsimony limit (0.949 = 14 steps in *P. nausithous*; 0.942 = 15 steps in *P. teleius*) led to a connection between the “Wolbachia” and the respective remaining haplotypes (Fig. 2). Thus, in both species there is a majority phylogroup plus several long branching groups, one of which is characterised by *Wolbachia* infection. Phylogenetic inference using both ML and MP yielded essentially the same results, with both *Phengaris teleius* and *P. nausithous* being monophyletic (Fig. 3, Fig. S2, Fig. S3). Together with *P*. *arion* the two species formed a clade clearly separated from other members of the genus. However, only in the parsimony analysis were *P*. *teleius* and *P. nausithous* supported as sister species (Fig. S3). In both species there is a basal “Wolbachia” clade sister to four (*P. teleius*) or two (*P. nausithous*) further haplogroups.

In *P. teleius* the majority of haplotypes formed a star-like network with many single steps (haplogroup *P. teleius* I) (Fig. 2). This phylogroup was distributed throughout continental Eurasia except for one haplotype which occurred in the most northern Japanese population (Hokkaido; ssp. *ogumae*) (Fig. 1). Three additional long-branched clades were geographically confined to Eastern Asia: *P. teleius* II to Honshū (ssp. *kazamoto* and *daisensis*), *P. teleius* III to Kyushu (ssp. *daisensis*) and *P. teleius* IV to China (ssp. *sinalcon*) and Japan (Hokkaido and Northern Honshū; ssp. *ogumae*, *kazamoto* and *hosonoi*). In the long branched *P. teleius* “Wolbachia” clade most individuals (94%; N = 15/16) were infected, significantly more than within the rest of *P. teleius* (2.8%; 4/141; X^2^-test: p<0.0001, Fig. 3). This clade was geographically restricted to Belarus, the Russian Altai, and Mongolia (Fig. 1). The subspecies within *P. teleius* showed no clear correspondence to haplogroups since subspecies either consisted of several haplotypes (ssp. *kazamoto, daisensis* and *ogumae*), or haplogroups harboured several subspecies (*P. teleius* I, II and IV).

In *P. nausithous* the majority haplogroup I was distributed through most of the species range. One additional clade was formed (*P. nausithous* II) by European haplotypes from Poland, Eastern Germany, Southern Germany, the Western Alps and Spain (Fig. 1, Fig. 2). In *P. nausithous* the “Wolbachia” clade harboured 56% infected individuals (N = 5/9) in contrast to the rest of *P. nausithous* (<1%; 1/110; X^2^-test: p<0.0001, Fig. 3). Infected individuals originated from Eastern Europe and Western Asia (Fig. 1). Subspecies *kijevensis* [41] had haplotypes of two clades, *P. nausithous* “Wolbachia” and *P. nausithous* I.

### Sequence Divergence and Nucleotide Diversity

Average sequence divergence between *Phengaris teleius* and *P. nausithous* was 4.19% ±0.54%, placing the split between the species at the end of the Pliocene or beginning of the Pleistocene (Table 1). In both species the sequence divergence between haplotypes of the “Wolbachia” clades and all other haplotypes was similar and translated into estimated ages between 0.65 and 1.97 myrs.

**Table 1:** Sequence divergence values and estimated node dates for prominent splits of recovered phylogenetic trees (Figure 3).

Split	Sequence divergence(%) between clades	MYA (Evolutionary rateof COI 1.3% per 1Million year)	MYA (Evolutionary rateof COI 2.3% per 1Million year)	MYA (Evolutionary rateof COI 3.5% per 1Million year)
*P. teleius* versus *P. nausithous*	4.19±0.54	3.22	1.82	1.19
*P. teleius* I-IV versus *P. teleius*“Wolbachia”	2.56±0.47	1.97	1.11	0.73
*P. teleius* IV versus *P. teleius* I-III	2.16±0.37	1.66	0.94	0.62
*P. teleius* II versus *P. teleius* I+III+IV	1.39±0.22	1.07	0.60	0.40
*P. teleius* III versus *P. teleius* I+II+IV	1.52±0.26	1.17	0.66	0.43
*P. nausithous* I-II versus *P. nausithous*“Wolbachia”	2.28±0.43	1.75	0.99	0.65
*P. nausithous* I versus *P. nausithous* II	1.42±0.30	1.09	0.62	0.41

Nucleotide diversity was π = 5.52 (including *Wolbachia* infected individuals: 7.73) for *P. teleius* and π = 5.41 (6.82) for *P. nausithous* (Table 2). Neutrality tests for different geographic areas revealed contrasting results for the two species. *P. teleius* showed low π combined with significantly negative Tajima’s *D* or Fu’s *F* for continental Asia and Europe suggestive of rapid demographic expansion in that area, whereas samples from Japan showed high π and no deviation from neutrality. In *P. nausithous*, clade *P. nausithous* I showed significant deviation from neutrality suggesting demographic expansion in the eastern part of the range, whereas in the western part *P. nausithous* II conformed to a neutral model.

**Table 2:** Nucleotide diversity *π*, Tajima’s *D*, and Fu’s *F* estimates at mtDNA COI of different phylogenetic clusters and geographic zones.

Sample pool	N	S	π ± s.d.	*Tajima*’*s D*	P value	*Fu*’*s F*	P value
*P. teleius* I+II+III+IV	141	88	5.52±2.96	−2.07	**0.002**	−25.18	**0.000**
*P. teleius* I+partly IV (Continental Eurasia)	117	57	2.56±1.53	−2.40	**0.000**	−26.83	**0.000**
*P. teleius* II+III+partly IV (Japan)	24	50	14.58±7.54	0.35	0.694	−00.71	0.414
*P. nausithous* I+II	110	40	5.41±2.91	−0.89	0.177	−20.01	**0.000**
*P. nausithous* I	76	33	2.21±1.37	−2.13	**0.002**	−27.07	**0.000**
*P. nausithous* II	34	12	2.52±1.55	−0.44	0.325	−01.03	0.330

N = number of sequences.

S = number of polymorphic sites.

### Nuclear Microsatellite Analysis

**Figure 4:**
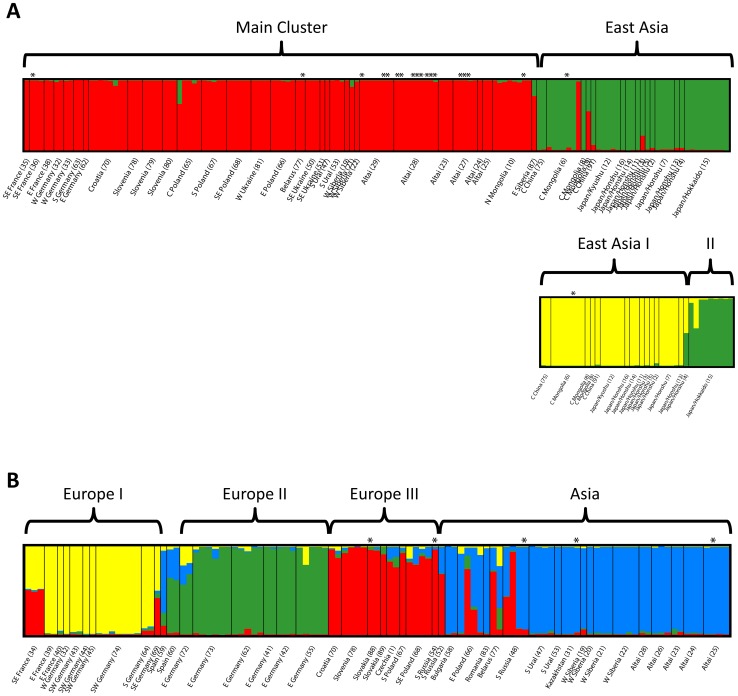
Results of STRUCTURE analysis of microsatellite genotypes for *Phengaris teleius* (A) and *P. nausithous* (B). Small bars represent individuals and their cluster membership coefficients. For details see text, Figure 1, and Figure S1; for population details see Table S1. *individuals infected with *Wolbachia.*

In *P. teleius*, the STRUCTURE analyses revealed consistent outcomes with *K* = 2, separating two geographically coherent clusters (Fig. 4, Fig. S1). The “Main Cluster” was formed by all samples from Europe and extended to continental Asia. The second cluster “East Asia” was formed by all samples from Japan, China and Central Mongolia. A few individuals showed admixture in the border region of the two clusters (Fig. 1). In additional separate STRUCTURE analyses of the two clusters the East Asian cluster was again split into two groups separating Hokkaido from the rest. Within the “Main Cluster” no further substructure was found as a peak of Δ*K* = 12 at *K* = 4 was very low compared to the other analyses (Fig. S1) and the resulting groups showed a high degree of admixture and no clear geographic pattern (data not shown). The “Main Cluster” in *P. teleius* corresponded largely to haplogroup *P. teleius* I obtained in the COI analysis, while the different Japanese clades and the “Wolbachia” clade were not retrieved in the microsatellite analysis.

In *P. nausithous* the STRUCTURE analysis revealed four clusters (Fig. 4, Fig. S1). Three clusters corresponded to areas in Europe (I, II, III) comprising western, central and eastern European populations, respectively. The fourth and largest cluster extended from Eastern Europe into Asia. Admixture was observed in contact zones of the clusters in specimens from France, Czech Republic, Belarus, Poland, SW Germany, and E Germany. Specimens from peripheral sites in Spain, Germany and Southern Russia also appeared admixed (Fig. 1). In STRUCTURE analyses at lower values of *K*, a strong East-West split was found. At *K* = 2, a western cluster comprising Europe I+II and an eastern cluster comprising Europe III+Asia was formed and at *K* = 3, Europe III was separated from Asia (data not shown).

Genetic differentiation between clusters was strong in both species (*P. teleius*: θ = 0.265 (SE 0.063), *G’_ST_ = *0.671; *P. nausithous*: θ = 0.143 (SE 0.02), *G’_ST_ = *0.497) although lower in *P. nausithous* as expected from the larger number of clusters. Genetic variation within clusters is shown in Table 3. In *P. teleius*, cluster East Asia I was the most genetically diverse as indicated by higher values of *H*_e_, *Ar* and private *Ar*. In *P. nausithous*, the four clusters showed similar levels of genetic variation.

**Table 3:** Mean estimates of genetic diversity across 8 microsatellite loci in clusters identified in the STRUCTURE analysis.

Species/cluster	N indivi-duals	*H*_e_ (SD)	*A*	*Ar* (SE)	private *Ar* (SE)	% private alleles
*P. teleius*						
Main Cluster	103	0.55 (0.26)	11.8	4.7 (1.0)	2.8 (1.0)	60%
East Asia I	26	0.67 (0.21)	9.0	5.4 (1.0)	3.3 (1.0)	61%
East Asia II	9	0.41 (0.31)	3.3	3.2 (0.7)	1.5 (0.7)	47%
*P. nausithous*						
Europe I	16	0.70 (0.23)	8.3	6.7 (1.5)	3.4 (1.4)	51%
Europe II	21	0.70 (0.24)	9.6	7.1 (1.0)	2.4 (0.6)	34%
Europe III	11	0.70 (0.23)	6.4	6.2 (1.3)	2.6 (1.0)	42%
Asia	37	0.71 (0.30)	13.9	7.8 (1.5)	3.5 (0.9)	45%

*H*_e_ expected heterozygosity, *A* mean number of alleles, *Ar* allelic richness based on 7 and 10 individuals, for *P. teleius* and *P. nausithous*, respectively.

Genetic divergence was largely consistent between nuclear and mitochondrial genomes, but influenced by the inclusion of *Wolbachia* haplogroups which did not form similarly divergent microsatellite clusters. In *P. teleius* genetic distances of microsatellites and COI sequences were not correlated when all haplotypes were considered (*r* = 0.083, Mantel-*p* = 0.12), but became significantly positively correlated when *Wolbachia* haplotypes were removed (*r* = 0.405, Mantel-*p* = 0.001). For *P. nausithous* genetic distances were correlated both overall (*r* = 0.269, Mantel-*p* = 0.001) and without *Wolbachia* haplotypes (*r* = 0.177, Mantel-*p* = 0.001).

## Discussion

### Phylogenetic Inference and *Wolbachia* Infection

Our phylogenetic analysis based on mtDNA COI sequences revealed that *P. teleius* and *P. nausithous* were clearly separated and formed well supported monophyletic clades. However, within both species we found highly distinct evolutionary lineages. These clades were not concordant with known subspecies nor did they represent spatially contiguous groups. Such an intraspecific phylogenetic pattern could be the result of either recent, secondary contact of formerly geographically separated populations of the species or it could be evidence for intrinsic reproductive barriers among sympatric cryptic species [83]. Indeed, the observed average sequence divergence between haplotypes of the “Wolbachia” clades and the rest of the species (2.28–2.56%) for *P. teleius* and *P. nausithous* resembled the divergence that has been reported between species [6]. Similar levels of divergence have already been found in *Phengaris teleius* and *P. nausithous* and have led to the hypothesis of cryptic species [44], [45]. However, the divergent haplogroups were strongly associated with *Wolbachia* infections in contrast to the remaining haplotypes. A similar pattern has been already described within other butterfly species [84], [85]. In fact, the COI sequences which led to the hypotheses of cryptic speciation within *P. teleius* ([45]: specimen Uk-08-J627) and within *P. nausithous* ([44]: specimen ZD-99-S301) corresponded perfectly to haplotypes that were associated with *Wolbachia* in our new samples originating from the same regions. This suggests that these specimens were also likely to be infected with *Wolbachia*. Our interpretation is corroborated by the fact that divergent haplotypes of infected and uninfected individuals co-occurred at several localities and that in both species the mtDNA *Wolbachia* clades were not reflected in the nuclear genome. Cryptic speciation should result in similar patterns across different genomes [28]. Thus, these inconsistencies between mitochondrial and nuclear data sets are evidence against cryptic species.

Our results suggest that the *Wolbachia* infection took place between 0.7–2.0 mya and 0.6–1.7 mya in *P. teleius* and *P. nausithous*, respectively, and well after species diversification, which we estimated between 1.2 and 3.2 mya, a time span consistent with previous estimates using external calibration points for chronology estimation [44]. However, the infection persists only in a minority of individuals from few populations. Hence, mitochondrial sequences of infected and uninfected parts of the populations accumulated substantial divergence, resulting in well separated clades in the inferred phylogeny. A similar phylogenetic pattern has been shown for other butterfly species [18], [85], [86], [87]. In 44% of the specimens of the *P. nausithous* “Wolbachia” clade no infection was detected. These individuals might indeed lack an infection, which can happen when *Wolbachia* is not efficiently transmitted to the next generation [88]. Thus, a negative *Wolbachia* test in particular samples does not disprove *Wolbachia* infection as causal for lineage divergence. However, we cannot exclude that the PCR-screening for *Wolbachia* might have produced false negatives, e.g. due to mutations in the primer binding sites.

Within 19 populations examined, *P. teleius* and *P. nausithous* co-occurred in the same locality. Three of these populations harbour a *Wolbachia* infection, either hosted by *P. teleius* (populations 28 and 77) or hosted by *P. nausithous* (population 25), but never hosted by both species at any locality. Our observation suggests low rates, or lack of horizontal transmission between the two sister species although there is general evidence from non-LTR retrotransposons for recent horizontal transmission between *Phengaris* species [89].

It has been shown that different *Wolbachia* strains can have different effects on the fitness of their hosts, ranging from positive to detrimental [90], [91]. In both species infected individuals were found across large parts of the distribution ranges from Belarus to Mongolia and from Slovakia to the South Ural Mountains, for *P. teleius* and *P. nausithous,* respectively. Because the *Wolbachia* infections were present within populations at low frequency in wide distributional areas infected individuals might experience a positive fitness effect due to the presence of *Wolbachia* [22]. Transmission rates into the next host generation seem to be imperfect, since the infection did not sweep through whole populations [22]. This effect could also depend on certain environmental conditions (e.g. *Wolbachia* density was highest in *Leptopilina* wasps at high temperatures, [23]). Indeed, in *P. teleius* only populations inhabiting steppe habitats with relatively hot and dry conditions in summer harboured *Wolbachia* infected individuals. Furthermore, in *P. teleius* two adults were *Wolbachia* infected both of which were male which might be an indication for a CI strain in *P. teleius* [11]. In *P. nausithous*, however, all four *Wolbachia* infected adults were females which might be an indication for a male-killing or feminization strain in that species [11]. For a better characteriziation of *Wolbachia* strains infecting *P*. *teleius* and *P. nausithous* and for clarification of its reproductive mechanisms and its fitness effects on hosts and populations further studies are needed, such as MLST genotyping [37], VNTR molecular screening [92], analyses of sex ratios and egg hatch-rates [19], or demographic models [87].

### Phylogeography of *Phengaris Teleius* and *P. nausithous*

In both species the samples that were not affected by *Wolbachia* showed considerable divergence in both the mitochondrial and the nuclear genome. However, the two species showed contrasting geographical patterns of differentiation and likely evolutionary scenarios.

In *P. teleius* there was little mtDNA variation across the western part of its range (*P. teleius* I). However, in East Asia three divergent haplogroups were found (*P. teleius* II, III, and IV). Although not fully concordant with the mtDNA pattern, the nuclear microsatellite data also revealed a stronger sub-structuring in Eastern Asia (East Asia I+II). This pattern might be well explained by the following scenario. After speciation of *P. teleius* between 1.2 and 3.2 mya, which likely took place in Central or Eastern Asia [40], [93], lineages may have spread and diversified throughout Eurasia. However, climatic conditions in one of the last glacial phases [94] could have eliminated the species from Europe and from most parts of continental Asia. In the Far East of continental Asia and Japan the species may have found larger or more suitable refugial areas [95], concordant with the high genetic diversity in that area. This phylogeographic hypothesis is also corroborated by the fact that all described subspecies in *P*. *teleius* are restricted to the Eastern Palaearctic and mainly to Japan [40]. Recolonization of continental Asia and Europe may then have started by founder individuals surviving in East Asia or Japan. The presence of isolated refugia in this area is likely given the complex topography and may be mirrored e.g. in the haplotypes T40, T41 and T42, or T02, forming distinct groups within *P*. *teleius* I (Fig. 2) and geographically confined to the Hustai Mountains in Central Mongolia (Pops. 6, 8, 9), or Hokkaido (Pop. 15). Low Tajima’s *D* and Fu’s *F* values coupled with the low nucleotide diversity (π) values of East Asia corroborate such a recent range expansion. Similar east-to-west colonization routes of butterflies have been suggested for an Eastern clade of *Melitaea cinxia* coming from Far Eastern populations and migrating into Scandinavia [96] as well as for *Coenonympha hero* which seems to have had a glacial centre of survival in the Southern Ural Mountains and expanded from there westwards to Europe [97]. However, although Eastern Asia clearly emerges as a centre of diversification within *P. teleius* in our analyses, the mtDNA phylogenetic clades and the microsatellite clusters were not congruent with the morphologically defined subspecies. Similar patterns were found e.g. in *Aglais urticae*, a nymphalid butterfly, and could be due to ecological differentiation occurring more rapidly than evolution at the mtDNA level [98].

In contrast to *P*. *teleius*, in *P. nausithous* the mitochondrial as well as the nuclear data set showed a stronger structuring of genetic diversity in the western part of its distribution. However, the divergence of the geographically separated haplogroups *P. nausithous* I and II was not concordant with the microsatellite clusters Europe I, II and III and their geographic patterns.

For *P. nausithous* a likely scenario is that after speciation in Central Asia it subsequently spread towards Western Asia and Europe where it diversified. Haplogroup *P. nausithous* II located in central and western Europe has slightly increased nucleotide diversity and non-significant Tajima’s *D* and Fu’s *F* values which suggest that the species survived during Pleistocene ice ages within European glacial refugia. For *P. nausithous* haplogroup I recent range expansion into the Eastern parts of its distribution range is likely to have started from a limited set of individuals indicated by significantly low Tajima’s *D* and Fu’s *F* values of Eurasian samples. The microsatellite clusters are also in line with the survival of *P. nausithous* in several European refugia. Three major European glacial refugia for animal species have been identified on the Iberian, Italian and Balkan peninsulas [94], [99], [100]. Cluster Asia and Europe III represent genetic groups that likely survived in the Balkans. Europe II represents a refugium located on the Iberian Peninsula and Europe I possibly represents a refugium on the Italian peninsular which today is not populated by the species anymore [42]. However, admixed populations in the contact zones of clusters Europe I and III, admixed populations in Spain and the distribution of cluster Europe III both east and west of the Alps indicate a complex phylogeographic history in western and central Europe. The East European (Europe III) and Asian clusters are overlapping as evidenced by several admixed populations located in East Poland, Belarus, and South Russia. Overall, *P. nausithous* shows complex phylogeographic patterns especially in contact zones and peripheral areas which deserve further analysis based on denser sampling.

## Conclusions

Based on mtDNA barcoding, nuclear microsatellite analyses and *Wolbachia* screening we reject the hypothesis of cryptic speciation within *Phengaris teleius* and *P. nausithous*. The major splits in the mtDNA phylogeny in both species can be explained by *Wolbachia* infections. Furthermore, geographic isolation during Pleistocene glaciations contributed to differentiation of mitochondrial and nuclear genomes.

Our study has shown that DNA barcoding studies can deliver robust information on cryptic species only in combination with tests for *Wolbachia* infections and additional analysis on nuclear markers, especially in groups with high prevalence of *Wolbachia-*infection [101].

Our study has some important implications for nature conservation. *Wolbachia* may constitute a risk for the stability of *Phengaris* populations, as an introduction of *Wolbachia* infected individuals into small populations might lead to a selective sweep and to full fixation of the associated introduced genotype within the population [22] which also means the elimination of the locally adapted genetic composition and diversity. Therefore, future reintroduction programs for insect species, like *Phengaris* butterflies, should include screening for the presence of *Wolbachia* in order not to introduce possibly detrimental elements into small and already threatened populations [87].

*P*. *teleius* showed increased genetic structuring in Eastern Asia whereas *P*. *nausithous* was more structured in Western Eurasia likely indicating opposing refugial areas during past glacial maxima. The phylogeographic clusters identified may represent locally adapted gene pools. Thus, reintroduction of the species into extinct European populations should include individuals from the same genetic group in order not to introduce possibly maladapted individuals. In *P. nausithous* four geographically separated microsatellite groups were observed in a small area in Europe. More detailed studies are necessary on the extent and delineation of these European clusters and the affiliation of peripheral populations.

## Supporting Information

Figure S1 **Determination of the most likely K of the STRUCTURE analyses.**
(DOC)

Figure S2 **Maximum Likelihood cladogram.**
(DOC)

Figure S3 **Maximum Parsimony cladogram.**
(DOC)

Table S1 ***Phengaris*** **material used for analysis.**
(DOC)

Table S2 **Microsatellite genotypes of** ***Phengaris teleius*** **and** ***P. nausithous*** **.**
(DOC)
