# Supplementary material for: Wolbachia Infections Mimic Cryptic Speciation in Two Parasitic Butterfly Species, Phengaris teleius and P. nausithous (Lepidoptera: Lycaenidae)
Source: PLoS One. 2013 Nov 6;8(11):e78107. doi: 10.1371/journal.pone.0078107 (PMC3819333; doi:10.1371/journal.pone.0078107)
Supplement: Figure S1 — Determination of the most likely K of the STRUCTURE analyses. (DOC) [file pone.0078107.s001.doc]

**Fig. S1** Determination of the most likely *K* of the STRUCTURE analyses according to . Mean values of Ln P(D) and Δ*K* as a function of *K* for different data sets of *P. teleius* and of *P. nausithous*

References:

1. Evanno G, Regnaut S, Goudet J (2005) Detecting the number of clusters of individuals using the software STRUCTURE: a simulation study. Mol Ecol 14: 2611-2620.
