# Supplementary material for: Wolbachia Infections Mimic Cryptic Speciation in Two Parasitic Butterfly Species, Phengaris teleius and P. nausithous (Lepidoptera: Lycaenidae)
Source: PLoS One. 2013 Nov 6;8(11):e78107. doi: 10.1371/journal.pone.0078107 (PMC3819333; doi:10.1371/journal.pone.0078107)
Supplement: Figure S2 — Maximum Likelihood cladogram. (DOC) [file pone.0078107.s002.doc]

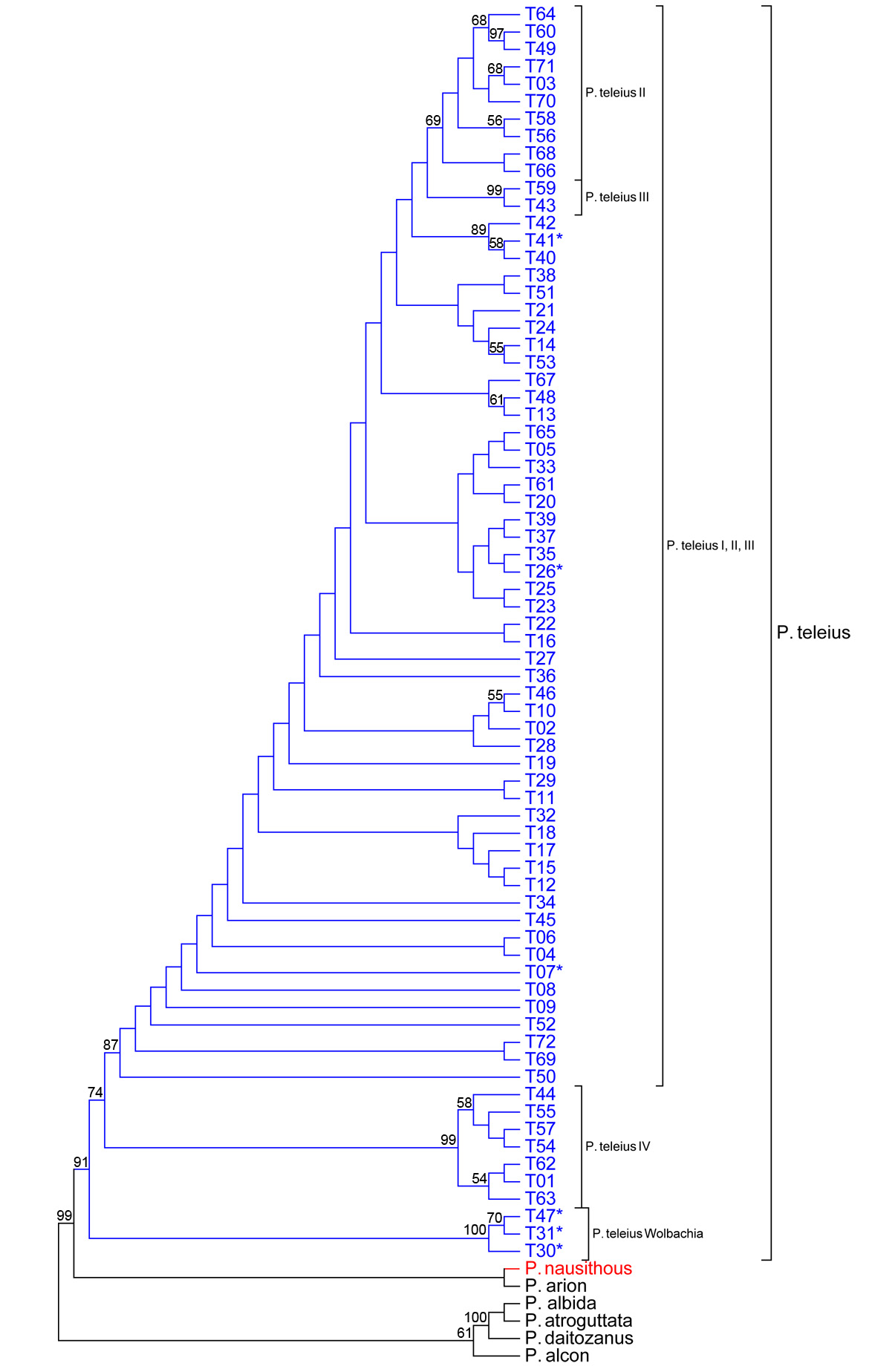


**Fig. S2a** ML cladogram using the TrN+I+G model of nucleotide substitution (-logL = 2105.87) and depicting relationships among haplotypes of *P*. *teleius* (blue). Haplotypes for *P*. *nausithous* (red) are collapsed. Bootstrap values in percent (>50%) are given. Origin of haplotypes according to Table S1; * haplotypes associated with *Wolbachia* infected individuals


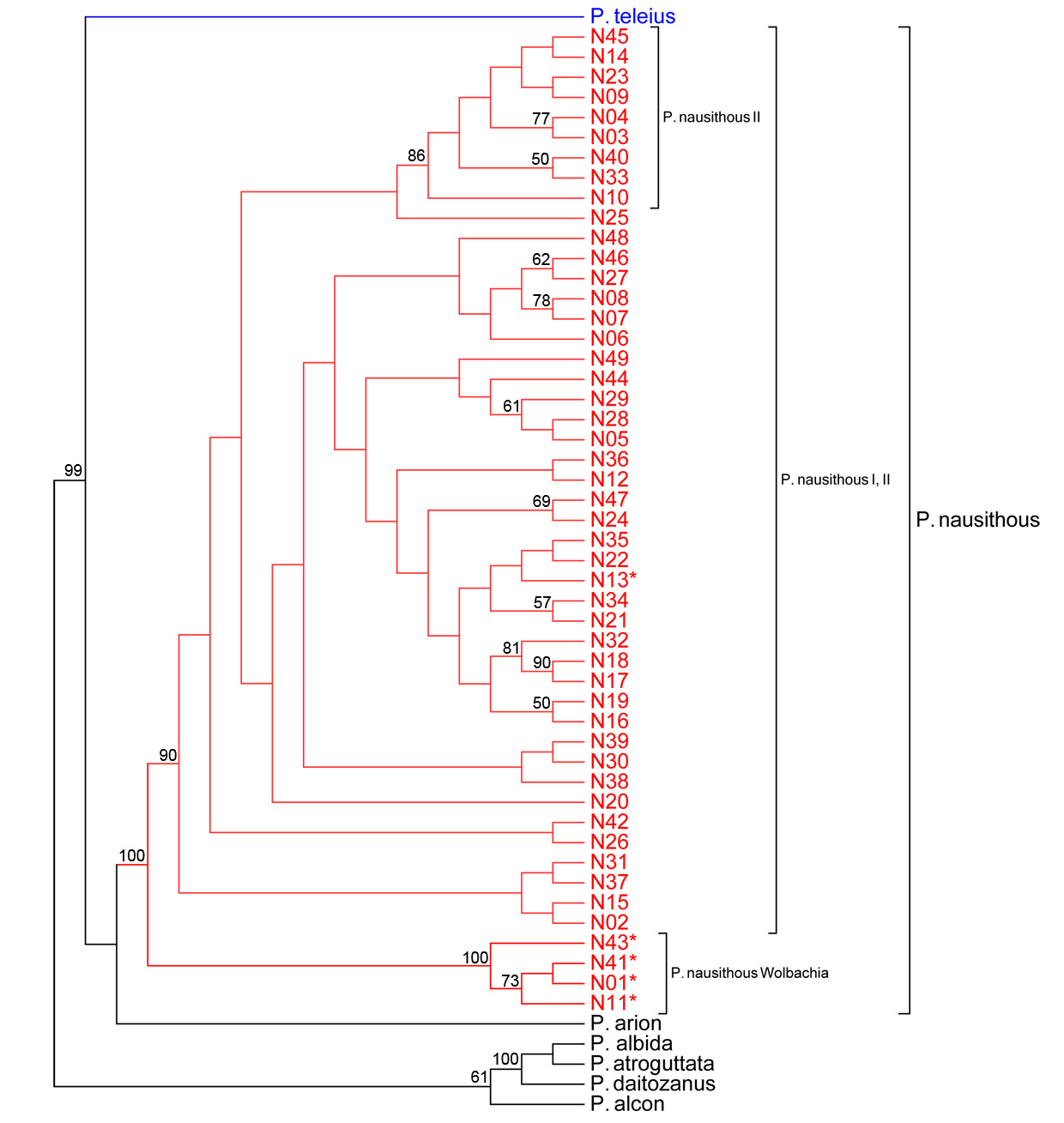


**Fig. S2b** ML cladogram using the TrN+I+G model of nucleotide substitution (-logL = 2105.87) and depicting relationships among haplotypes of *P*. *nausithous* (red). Haplotypes for *P*. *teleius* (blue) are collapsed. Bootstrap values in percent (>50%) are given. Origin of haplotypes according to Table S1; * haplotypes associated with *Wolbachia* infected individuals
