# Supplementary material for: Wolbachia Infections Mimic Cryptic Speciation in Two Parasitic Butterfly Species, Phengaris teleius and P. nausithous (Lepidoptera: Lycaenidae)
Source: PLoS One. 2013 Nov 6;8(11):e78107. doi: 10.1371/journal.pone.0078107 (PMC3819333; doi:10.1371/journal.pone.0078107)
Supplement: Figure S3 — Maximum Parsimony cladogram. (DOC) [file pone.0078107.s003.doc]

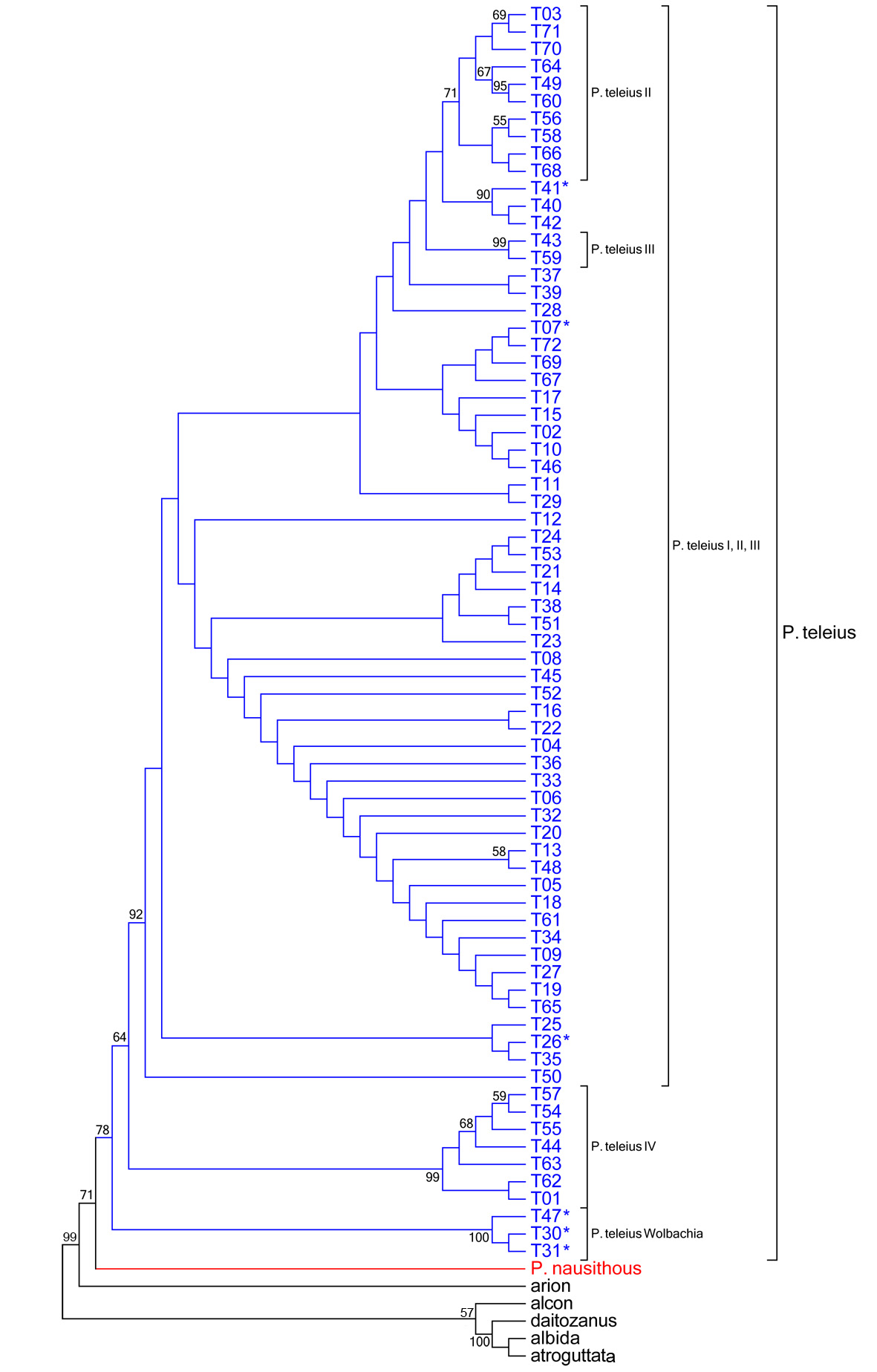


**Fig. S3a** Cladogram of tree #1 out of 131 most parsimonious trees (length = 687) depicting relationships among haplotypes of *P*. *teleius* (blue). Haplotypes for *P*. *nausithous* (red) are collapsed. Bootstrap values in percent (>50%) are given. Origin of haplotypes according to Table S1; * haplotypes associated with *Wolbachia* infected individuals


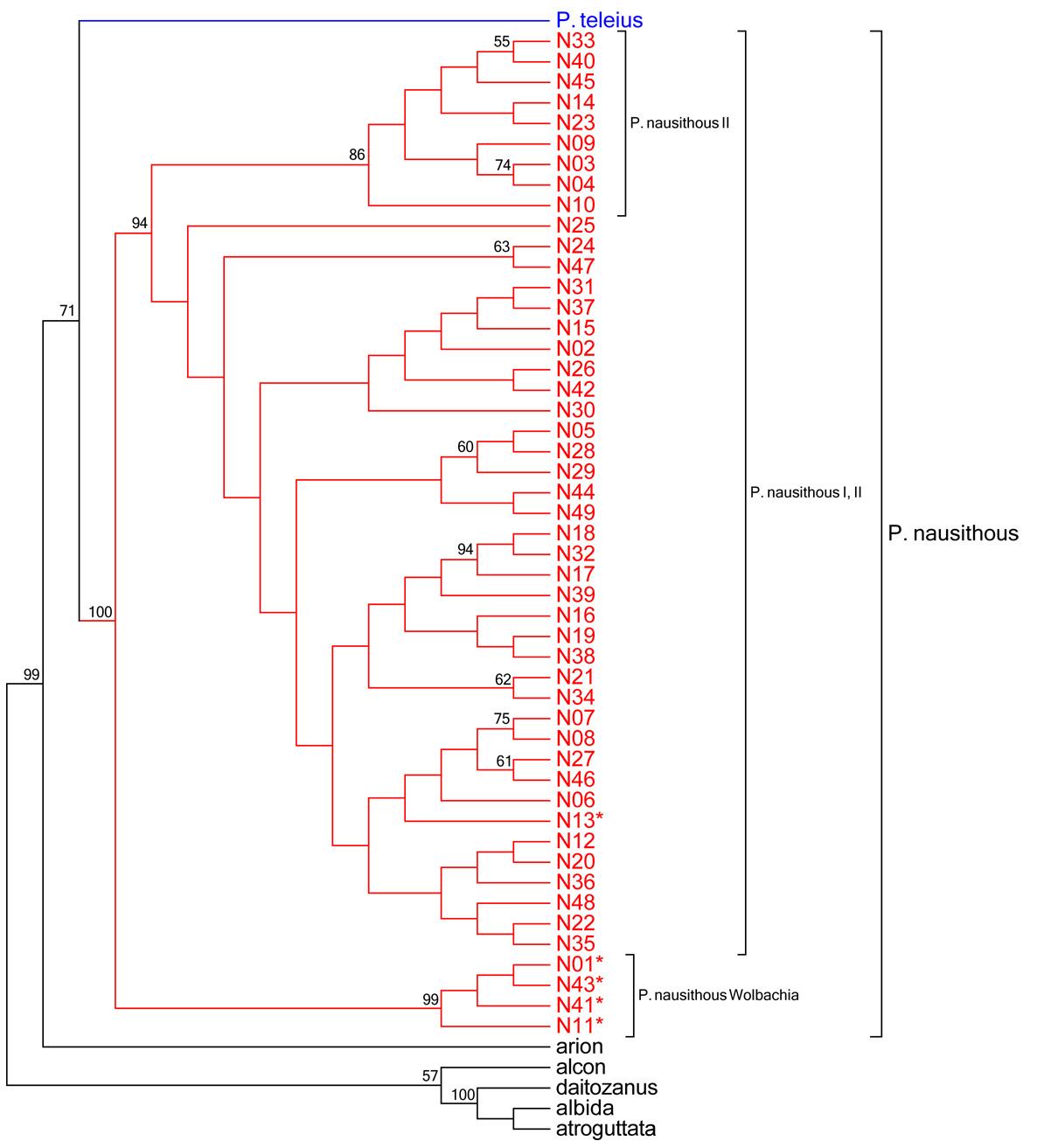


**Fig. S3b** Cladogram of tree #1 out of 131 most parsimonious trees (length = 687) depicting relationships among haplotypes of *P*. *nausithous* (red). Haplotypes for *P*. *teleius* (blue) are collapsed. Bootstrap values in percent (>50%) are given. Origin of haplotypes according to Table S1; * haplotypes associated with *Wolbachia* infected individuals
