# Supplementary material for: Wolbachia Infections Mimic Cryptic Speciation in Two Parasitic Butterfly Species, Phengaris teleius and P. nausithous (Lepidoptera: Lycaenidae)
Source: PLoS One. 2013 Nov 6;8(11):e78107. doi: 10.1371/journal.pone.0078107 (PMC3819333; doi:10.1371/journal.pone.0078107)
Supplement: Table S1 — Phengaris material used for analysis. (DOC) [file pone.0078107.s004.doc]

**Table S1** *Phengaris* material used for analysis

| Specimen voucher | Species | Collecting locality | Latitude | Longitude |  | Pop  code | Sex | w+ | Haplotypes | COI clade | GenBank  accession numbers | Source |
| --- | --- | --- | --- | --- | --- | --- | --- | --- | --- | --- | --- | --- |
| SR006.01 | *P. teleius* *euphemia* | C Mongolia, Hustai mountains | 47°38’N | 105°52’E |  | 06 |  | no | **T41** | *P. teleius* I | JX311049 | This paper |
| SR006.03 | *P. teleius* *euphemia* | C Mongolia, Hustai mountains | 47°38’N | 105°52’E |  | 06 |  | no | **T41** | *P. teleius* I | JX311050 | This paper |
| SR006.05 | *P. teleius* *euphemia* | C Mongolia, Hustai mountains | 47°38’N | 105°52’E |  | 06 |  | no | **T42** | *P. teleius* I | JX311051 | This paper |
| SR006.06 | *P. teleius* *euphemia* | C Mongolia, Hustai mountains | 47°38’N | 105°52’E |  | 06 | ♂ | yes | **T41** | *P. teleius* I | JX311052 | This paper |
| SR006.07 | *P. teleius* *euphemia* | C Mongolia, Hustai mountains | 47°38’N | 105°52’E |  | 06 |  | no | **T41** | *P. teleius* I | JX311053 | This paper |
| SR006.08 | *P. teleius* *euphemia* | C Mongolia, Hustai mountains | 47°38’N | 105°52’E |  | 06 |  | no | **T41** | *P. teleius* I | JX311054 | This paper |
| SR006.10 | *P. teleius* *euphemia* | C Mongolia, Hustai mountains | 47°38’N | 105°52’E |  | 06 |  | no | **T07** | *P. teleius* I | JX311055 | This paper |
| SR008.01 | *P. teleius* *euphemia* | C Mongolia, Hustai mountains | 47°45’N | 105°53’E |  | 08 |  | no | **T40** | *P. teleius* I | JX311056 | This paper |
| SR009.01 | *P. teleius* *euphemia* | C Mongolia, Hustai mountains | 47°44’N | 105°52’E |  | 09 |  | no | **T37** | *P. teleius* I | JX311057 | This paper |
| SR010.01 | *P. teleius* *euphemia* | N Mongolia, Chentej mountains | 49°05’N | 107°17’E |  | 10 |  | no | **T07** | *P. teleius* I | JX311058 | This paper |
| SR010.14 | *P. teleius* *euphemia* | N Mongolia, Chentej mountains | 49°05’N | 107°17’E |  | 10 |  | no | **T35** | *P. teleius* I | JX311059 | This paper |
| SR010.15 | *P. teleius* *euphemia* | N Mongolia, Chentej mountains | 49°05’N | 107°17’E |  | 10 |  | no | **T39** | *P. teleius* I | JX311060 | This paper |
| SR010.17 | *P. teleius* *euphemia* | N Mongolia, Chentej mountains | 49°05’N | 107°17’E |  | 10 |  | yes | **T30** | *P. teleius* Wolbachia | JX311061 | This paper |
| SR010.20 | *P. teleius* *euphemia* | N Mongolia, Chentej mountains | 49°05’N | 107°17’E |  | 10 |  | no | **T38** | *P. teleius* I | JX311062 | This paper |
| SR010.21 | *P. teleius* *euphemia* | N Mongolia, Chentej mountains | 49°05’N | 107°17’E |  | 10 |  | no | **T38** | *P. teleius* I | JX311063 | This paper |
| SR010.23 | *P. teleius* *euphemia* | N Mongolia, Chentej mountains | 49°05’N | 107°17’E |  | 10 |  | yes | **T30** | *P. teleius* Wolbachia | JX311064 | This paper |
| SR010.32 | *P. teleius* *euphemia* | N Mongolia, Chentej mountains | 49°05’N | 107°17’E |  | 10 |  | no | **T37** | *P. teleius* I | JX311065 | This paper |
| SR010.38 | *P. teleius* *euphemia* | N Mongolia, Chentej mountains | 49°05’N | 107°17’E |  | 10 |  | no | **T36** | *P. teleius* I | JX311066 | This paper |
| SR010.46 | *P. teleius* *euphemia* | N Mongolia, Chentej mountains | 49°05’N | 107°17’E |  | 10 |  | no | **T35** | *P. teleius* I | JX311067 | This paper |
| SR019.21 | *P. teleius* *obscurata* ^#^ | Russia, Novosibirsk, near Akademgorodok | 54°49’N | 83°06’E |  | 19 |  | no | **T34** | *P. teleius* I | JX311069 | This paper |
| SR021.44 | *P. teleius* *obscurata* ^#^ | Russia, Novosibirsk, near Akademgorodok | 54°50’N | 83°08’E |  | 21 |  | no | **T07** | *P. teleius* I | JX311075 | This paper |
| SR022.04 | *P. teleius* *obscurata* ^#^ | Russia, near Barnaul | 53°22’N | 84°03’E |  | 22 |  | no | **T07** | *P. teleius* I | JX311078 | This paper |
| SR023.09 | *P. teleius* *obscurata* ^#^ | Russia, Altai mountains, around Cherga | 51°33’N | 85°33’E |  | 23 |  | no | **T33** | *P. teleius* I | JX311083 | This paper |
| SR023.10 | *P. teleius* *obscurata* ^#^ | Russia, Altai mountains, around Cherga | 51°33’N | 85°33’E |  | 23 |  | no | **T07** | *P. teleius* I | JX311084 | This paper |
| SR023.33 | *P. teleius* *obscurata* ^#^ | Russia, Altai mountains, around Cherga | 51°33’N | 85°33’E |  | 23 |  | no | **T33** | *P. teleius* I | JX311086 | This paper |
| SR024.01 | *P. teleius* *obscurata* ^#^ | Russia, Altai mountains, Choya-District, near Levinka | 51°57’N | 86°23’E |  | 24 |  | no | **T48** | *P. teleius* I | JX311087 | This paper |
| SR025.04 | *P. teleius* *obscurata* ^#^ | Russia, Altai mountains, Turochak District, near Verkh Biisk | 52°02’N | 87°04’E |  | 25 |  | no | **T32** | *P. teleius* I | JX311093 | This paper |
| SR025.12 | *P. teleius* *obscurata* ^#^ | Russia, Altai mountains, Turochak District, near Verkh Biisk | 52°02’N | 87°04’E |  | 25 |  | no | **T32** | *P. teleius* I | JX311096 | This paper |
| SR027.01 | *P. teleius* *obscurata* ^#^ | Russia, Altai mountains, Ongutai District, near Kurota | 50°49’N | 85°60’E |  | 27 |  | no | **T07** | *P. teleius* I | JX311100 | This paper |
| SR027.03 | *P. teleius* *obscurata* ^#^ | Russia, Altai mountains, Ongutai District, near Kurota | 50°49’N | 85°60’E |  | 27 |  | yes | **T07** | *P. teleius* I | JX311101 | This paper |
| SR027.04 | *P. teleius* *obscurata* ^#^ | Russia, Altai mountains, Ongutai District, near Kurota | 50°49’N | 85°60’E |  | 27 |  | yes | **T31** | *P. teleius* Wolbachia | JX311102 | This paper |
| SR027.08 | *P. teleius* *obscurata* ^#^ | Russia, Altai mountains, Ongutai District, near Kurota | 50°49’N | 85°60’E |  | 27 |  | yes | **T30** | *P. teleius* Wolbachia | JX311103 | This paper |
| SR027.12 | *P. teleius* *obscurata* ^#^ | Russia, Altai mountains, Ongutai District, near Kurota | 50°49’N | 85°60’E |  | 27 |  | no | **T07** | *P. teleius* I | JX311104 | This paper |
| SR028.01 | *P. teleius* *obscurata* ^#^ | Russia, Altai mountains, Shebalino District, near Ilyianka | 51°20’N | 85°10’E |  | 28 |  | yes | **T30** | *P. teleius* Wolbachia | JX311105 | This paper |
| SR028.02 | *P. teleius* *obscurata* ^#^ | Russia, Altai mountains, Shebalino District, near Ilyianka | 51°20’N | 85°10’E |  | 28 |  | yes | **T30** | *P. teleius* Wolbachia | JX311106 | This paper |
| SR028.07 | *P. teleius* *obscurata* ^#^ | Russia, Altai mountains, Shebalino District, near Ilyianka | 51°20’N | 85°10’E |  | 28 |  | no | **T29** | *P. teleius* I | JX311107 | This paper |
| SR028.15 | *P. teleius* *obscurata* ^#^ | Russia, Altai mountains, Shebalino District, near Ilyianka | 51°20’N | 85°10’E |  | 28 |  | yes | **T30** | *P. teleius* Wolbachia | JX311108 | This paper |
| SR028.16 | *P. teleius* *obscurata* ^#^ | Russia, Altai mountains, Shebalino District, near Ilyianka | 51°20’N | 85°10’E |  | 28 |  | yes | **T30** | *P. teleius* Wolbachia | JX311109 | This paper |
| SR028.18 | *P. teleius* *obscurata* ^#^ | Russia, Altai mountains, Shebalino District, near Ilyianka | 51°20’N | 85°10’E |  | 28 |  | yes | **T30** | *P. teleius* Wolbachia | JX311110 | This paper |
| SR028.21 | *P. teleius* *obscurata* ^#^ | Russia, Altai mountains, Shebalino District, near Ilyianka | 51°20’N | 85°10’E |  | 28 |  | yes | **T30** | *P. teleius* Wolbachia | JX311111 | This paper |
| SR028.26 | *P. teleius* *obscurata* ^#^ | Russia, Altai mountains, Shebalino District, near Ilyianka | 51°20’N | 85°10’E |  | 28 |  | yes | **T30** | *P. teleius* Wolbachia | JX311113 | This paper |
| SR028.28 | *P. teleius* *obscurata* ^#^ | Russia, Altai mountains, Shebalino District, near Ilyianka | 51°20’N | 85°10’E |  | 28 |  | yes | **T30** | *P. teleius* Wolbachia | JX311114 | This paper |
| SR029.03 | *P. teleius* *obscurata* ^#^ | Russia, Altai mountains, Kyrlyk river, near Kyrlyk | 50°42’N | 84°58’E |  | 29 |  | yes | **T30** | *P. teleius* Wolbachia | JX311116 | This paper |
| SR029.05 | *P. teleius* *obscurata* ^#^ | Russia, Altai mountains, Kyrlyk river, near Kyrlyk | 50°42’N | 84°58’E |  | 29 |  | no | **T29** | *P. teleius* I | JX311117 | This paper |
| SR029.07 | *P. teleius* *obscurata* ^#^ | Russia, Altai mountains, Kyrlyk river, near Kyrlyk | 50°42’N | 84°58’E |  | 29 |  | no | **T29** | *P. teleius* I | JX311118 | This paper |
| SR029.09 | *P. teleius* *obscurata* ^#^ | Russia, Altai mountains, Kyrlyk river, near Kyrlyk | 50°42’N | 84°58’E |  | 29 |  | no | **T29** | *P. teleius* I | JX311119 | This paper |
| SR029.10 | *P. teleius* *obscurata* ^#^ | Russia, Altai mountains, Kyrlyk river, near Kyrlyk | 50°42’N | 84°58’E |  | 29 |  | no | **T07** | *P. teleius* I | JX311120 | This paper |
| SR029.12 | *P. teleius* *obscurata* ^#^ | Russia, Altai mountains, Kyrlyk river, near Kyrlyk | 50°42’N | 84°58’E |  | 29 |  | yes | **T30** | *P. teleius* Wolbachia | JX311121 | This paper |
| SR029.13 | *P. teleius* *obscurata* ^#^ | Russia, Altai mountains, Kyrlyk river, near Kyrlyk | 50°42’N | 84°58’E |  | 29 |  | no | **T29** | *P. teleius* I | JX311122 | This paper |
| SR032.01 | *P. teleius* *teleius* | Germany, Westerwald, around Montabaur | 50°25’N | 07°48’E |  | 32 |  | no | **T07** | *P. teleius* I | JX311126 | This paper |
| SR032.03 | *P. teleius* *teleius* | Germany, Westerwald, around Montabaur | 50°25’N | 07°48’E |  | 32 |  | no | **T52** | *P. teleius* I | JX311128 | This paper |
| SR033.01 | *P. teleius* *teleius* | Germany, Westerwald, around Montabaur | 50°29’N | 07°55’E |  | 33 |  | no | **T28** | *P. teleius* I | JX311129 | This paper |
| SR033.02 | *P. teleius* *teleius* | Germany, Westerwald, around Montabaur | 50°29’N | 07°55’E |  | 33 |  | no | **T07** | *P. teleius* I | JX311130 | This paper |
| SR035.01 | *P. teleius* *teleius* | France, Rhône-Alpes, Divonne-les-Bains, Ain | 46°22’N | 06°10’E | * | 35 |  | no | **T25** | *P. teleius* I | JX311134 | This paper |
| SR036.01 | *P. teleius* *teleius* | France, Rhône-Alpes, Ceyzérieu, Ain | 45°50’N | 05°45’E | * | 36 |  | yes | **T26** | *P. teleius* I | JX311135 | This paper |
| SR036.02 | *P. teleius* *teleius* | France, Rhône-Alpes, Ceyzérieu, Ain | 45°50’N | 05°45’E | * | 36 |  | no | **T27** | *P. teleius* I | JX311136 | This paper |
| SR036.04 | *P. teleius* *teleius* | France, Rhône-Alpes, Ceyzérieu, Ain | 45°50’N | 05°45’E | * | 36 |  | no | **T26** | *P. teleius* I | JX311137 | This paper |
| SR038.01 | *P. teleius* *teleius* | France, Lorraine, Vergonge, Jolivet | 48°36’N | 06°30’E | * | 38 |  | no | **T25** | *P. teleius* I | JX311138 | This paper |
| SR038.03 | *P. teleius* *teleius* | France, Lorraine, Vergonge, Jolivet | 48°36’N | 06°30’E | * | 38 |  | no | **T25** | *P. teleius* I | JX311139 | This paper |
| SR047.19 | *P. teleius* *obscurata* ^#^ | Russia, S Ural mountains, Cheljabinsk region, near Snezhinsk | 56°05’N | 60°44’E | * | 47 |  | no | **T24** | *P. teleius* I | JX311157 | This paper |
| SR050.01 | *P. teleius* *teleius* | SE Ukraine, Kharkov region, near Mohnach | 49°59’N | 36°12’E | * | 50 |  | no | **T05** | *P. teleius* I | JX311162 | This paper |
| SR050.03 | *P. teleius* *teleius* | SE Ukraine, Kharkov region, near Mohnach | 49°59’N | 36°12’E | * | 50 |  | no | **T05** | *P. teleius* I | JX311163 | This paper |
| SR050.05 | *P. teleius* *teleius* | SE Ukraine, Kharkov region, near Mohnach | 49°59’N | 36°12’E | * | 50 |  | no | **T07** | *P. teleius* I | JX311164 | This paper |
| SR051.01 | *P. teleius* *teleius* | SE Ukraine, Lugansk region, near Ilienko | 48°34’N | 39°18’E | * | 51 |  | no | **T05** | *P. teleius* I | JX311165 | This paper |
| SR053.01 | *P. teleius* *obscurata* ^#^ | Russia, S Ural mountains, Cheljabinsk region, Itkul lake | 56°21’N | 62°11’E | * | 53 |  | no | **T53** | *P. teleius* I | JX311167 | This paper |
| SR053.02 | *P. teleius* *obscurata* ^#^ | Russia, S Ural mountains, Cheljabinsk region, Itkul lake | 56°21’N | 62°11’E | * | 53 |  | no | **T07** | *P. teleius* I | JX311168 | This paper |
| SR053.03 | *P. teleius* *obscurata* ^#^ | Russia, S Ural mountains, Cheljabinsk region, Itkul lake | 56°21’N | 62°11’E | * | 53 |  | no | **T07** | *P. teleius* I | JX311169 | This paper |
| SR062.01 | *P. teleius* *teleius* | Germany, Saxony, near Schkeuditz | 51°23’N | 12°11’E |  | 62 |  | no | **T51** | *P. teleius* I | JX311182 | This paper |
| SR062.04 | *P. teleius* *teleius* | Germany, Saxony, near Schkeuditz | 51°23’N | 12°11’E |  | 62 |  | no | **T51** | *P. teleius* I | JX311183 | This paper |
| SR063.01 | *P. teleius* *teleius* | Germany, Bavaria, Lake Constance, near Constance | 47°39’N | 09°10’E | * | 63 |  | no | **T23** | *P. teleius* I | JX311189 | This paper |
| SR063.03 | *P. teleius* *teleius* | Germany, Bavaria, Lake Constance, near Constance | 47°39’N | 09°10’E | * | 63 |  | no | **T23** | *P. teleius* I | JX311190 | This paper |
| SR065.02 | *P. teleius* *teleius* | C Poland, Wolka | 52°16’N | 20°42’E |  | 65 |  | no | **T22** | *P. teleius* I | JX311193 | This paper |
| SR065.03 | *P. teleius* *teleius* | C Poland, Wolka | 52°16’N | 20°42’E |  | 65 |  | no | **T22** | *P. teleius* I | JX311194 | This paper |
| SR065.04 | *P. teleius* *teleius* | C Poland, Wolka | 52°16’N | 20°42’E |  | 65 |  | no | **T22** | *P. teleius* I | JX311195 | This paper |
| SR065.05 | *P. teleius* *teleius* | C Poland, Wolka | 52°16’N | 20°42’E |  | 65 |  | no | **T22** | *P. teleius* I | JX311196 | This paper |
| SR065.06 | *P. teleius* *teleius* | C Poland, Wolka | 52°16’N | 20°42’E |  | 65 |  | no | **T21** | *P. teleius* I | JX311197 | This paper |
| SR066.02 | *P. teleius* *teleius* | E Poland, Kosyn | 51°23’N | 23°34’E |  | 66 |  | no | **T65** | *P. teleius* I | JX311198 | This paper |
| SR066.03 | *P. teleius* *teleius* | E Poland, Kosyn | 51°23’N | 23°34’E |  | 66 |  | no | **T07** | *P. teleius* I | JX311199 | This paper |
| SR066.04 | *P. teleius* *teleius* | E Poland, Kosyn | 51°23’N | 23°34’E |  | 66 |  | no | **T19** | *P. teleius* I | JX311200 | This paper |
| SR066.05 | *P. teleius* *teleius* | E Poland, Kosyn | 51°23’N | 23°34’E |  | 66 |  | no | **T20** | *P. teleius* I | JX311201 | This paper |
| SR066.06 | *P. teleius* *teleius* | E Poland, Kosyn | 51°23’N | 23°34’E |  | 66 |  | no | **T19** | *P. teleius* I | JX311202 | This paper |
| SR067.02 | *P. teleius* *teleius* | S Poland, Wiesiółka | 50°25’N | 19°21’E |  | 67 |  | no | **T18** | *P. teleius* I | JX311207 | This paper |
| SR067.03 | *P. teleius* *teleius* | S Poland, Wiesiółka | 50°25’N | 19°21’E |  | 67 |  | no | **T17** | *P. teleius* I | JX311208 | This paper |
| SR067.04 | *P. teleius* *teleius* | S Poland, Wiesiółka | 50°25’N | 19°21’E |  | 67 |  | no | **T17** | *P. teleius* I | JX311209 | This paper |
| SR067.05 | *P. teleius* *teleius* | S Poland, Wiesiółka | 50°25’N | 19°21’E |  | 67 |  | no | **T16** | *P. teleius* I | JX311210 | This paper |
| SR067.06 | *P. teleius* *teleius* | S Poland, Wiesiółka | 50°25’N | 19°21’E |  | 67 |  | no | **T16** | *P. teleius* I | JX311211 | This paper |
| SR068.02 | *P. teleius* *teleius* | SE Poland, Widacz | 49°38’N | 21°50’E |  | 68 |  | no | **T07** | *P. teleius* I | JX311213 | This paper |
| SR068.03 | *P. teleius* *teleius* | SE Poland, Widacz | 49°38’N | 21°50’E |  | 68 |  | no | **T15** | *P. teleius* I | JX311214 | This paper |
| SR068.04 | *P. teleius* *teleius* | SE Poland, Widacz | 49°38’N | 21°50’E |  | 68 |  | no | **T07** | *P. teleius* I | JX311215 | This paper |
| SR068.05 | *P. teleius* *teleius* | SE Poland, Widacz | 49°38’N | 21°50’E |  | 68 |  | no | **T15** | *P. teleius* I | JX311216 | This paper |
| SR068.06 | *P. teleius* *teleius* | SE Poland, Widacz | 49°38’N | 21°50’E |  | 68 |  | no | **T14** | *P. teleius* I | JX311217 | This paper |
| SR070.02 | *P. teleius* *teleius* | Croatia, Bedekovičeve Grabe | 46°26’N | 16°24’E | * | 70 |  | no | **T13** | *P. teleius* I | JX311223 | This paper |
| SR070.08 | *P. teleius* *teleius* | Croatia, Bedekovičeve Grabe | 46°26’N | 16°24’E | * | 70 |  | no | **T07** | *P. teleius* I | JX311225 | This paper |
| SR070.09 | *P. teleius* *teleius* | Croatia, Bedekovičeve Grabe | 46°26’N | 16°24’E | * | 70 |  | no | **T12** | *P. teleius* I | JX311226 | This paper |
| SR070.13 | *P. teleius* *teleius* | Croatia, Bedekovičeve Grabe | 46°26’N | 16°24’E | * | 70 |  | no | **T46** | *P. teleius* I | JX311227 | This paper |
| SR070.16 | *P. teleius* *teleius* | Croatia, Bedekovičeve Grabe | 46°26’N | 16°24’E | * | 70 |  | no | **T11** | *P. teleius* I | JX311229 | This paper |
| SR070.19 | *P. teleius* *teleius* | Croatia, Bedekovičeve Grabe | 46°26’N | 16°24’E | * | 70 |  | no | **T46** | *P. teleius* I | JX311230 | This paper |
| SR070.28 | *P. teleius* *teleius* | Croatia, Bedekovičeve Grabe | 46°26’N | 16°24’E | * | 70 |  | no | **T10** | *P. teleius* I | JX311231 | This paper |
| SR070.31 | *P. teleius* *teleius* | Croatia, Bedekovičeve Grabe | 46°26’N | 16°24’E | * | 70 |  | no | **T61** | *P. teleius* I | JX311232 | This paper |
| SR075.04 | *P. teleius* *sinalcon* | China, SW Gansu, Qinling mountains, valley SE of Bola (Hezuo) | 33°55’N | 101°40’E | * | 75 |  | no | **T63** | *P .teleius* IV | JX311248 | This paper |
| SR075.08 | *P. teleius* *sinalcon* | China, SW Gansu, Qinling mountains, valley SE of Bola (Hezuo) | 33°55’N | 101°40’E | * | 75 |  | no | **T62** | *P. teleius* IV | JX311249 | This paper |
| SR077.02 | *P. teleius* *teleius* | Belarus | 51°48’N | 30°15’E | * | 77 |  | no | **T50** | *P. teleius* Wolbachia | JX311250 | This paper |
| SR077.03 | *P. teleius* *teleius* | Belarus | 51°48’N | 30°15’E | * | 77 | ♂ | yes | **T47** | *P. teleius* Wolbachia | JX311251 | This paper |
| SR078.02 | *P. teleius* *teleius* | Slovenia, Celje | 46°14’N | 15°15’E | * | 78 |  | no | **T09** | *P. teleius* I | JX311254 | This paper |
| SR078.03 | *P. teleius* *teleius* | Slovenia, Celje | 46°14’N | 15°15’E | * | 78 |  | no | **T07** | *P. teleius* I | JX311255 | This paper |
| SR078.10 | *P. teleius* *teleius* | Slovenia, Celje | 46°14’N | 15°15’E | * | 78 |  | no | **T45** | *P. teleius* I | JX311260 | This paper |
| SR079.03 | *P. teleius* *teleius* | Slovenia, Ilirska Bistrica | 45°34’N | 14°14’E | * | 79 |  | no | **T07** | *P. teleius* I | JX311261 | This paper |
| SR079.04 | *P. teleius* *teleius* | Slovenia, Ilirska Bistrica | 45°34’N | 14°14’E | * | 79 |  | no | **T07** | *P. teleius* I | JX311262 | This paper |
| SR079.06 | *P. teleius* *teleius* | Slovenia, Ilirska Bistrica | 45°34’N | 14°14’E | * | 79 |  | no | **T07** | *P. teleius* I | JX311263 | This paper |
| SR079.07 | *P. teleius* *teleius* | Slovenia, Ilirska Bistrica | 45°34’N | 14°14’E | * | 79 |  | no | **T07** | *P. teleius* I | JX311264 | This paper |
| SR080.01 | *P. teleius* *teleius* | Slovenia, Nova Gorika | 45°57’N | 13°39’E | * | 80 |  | no | **T07** | *P. teleius* I | JX311265 | This paper |
| SR080.02 | *P. teleius* *teleius* | Slovenia, Nova Gorika | 45°57’N | 13°39’E | * | 80 |  | no | **T07** | *P. teleius* I | JX311266 | This paper |
| SR080.03 | *P. teleius* *teleius* | Slovenia, Nova Gorika | 45°57’N | 13°39’E | * | 80 |  | no | **T08** | *P. teleius* I | JX311267 | This paper |
| SR080.05 | *P. teleius* *teleius* | Slovenia, Nova Gorika | 45°57’N | 13°39’E | * | 80 |  | no | **T07** | *P. teleius* I | JX311268 | This paper |
| SR081.02 | *P. teleius* *teleius* | Ukraine, Transcarpathia, Mukachero District | 48°26’N | 22°43’E | * | 81 |  | no | **T04** | *P. teleius* I | JX311269 | This paper |
| SR081.06 | *P. teleius* *teleius* | Ukraine, Transcarpathia, Mukachero District | 48°26’N | 22°43’E | * | 81 |  | no | **T07** | *P. teleius* I | JX311270 | This paper |
| SR081.09 | *P. teleius* *teleius* | Ukraine, Transcarpathia, Mukachero District | 48°26’N | 22°43’E | * | 81 |  | no | **T06** | *P. teleius* I | JX311271 | This paper |
| SR081.13 | *P. teleius* *teleius* | Ukraine, Transcarpathia, Mukachero District | 48°26’N | 22°43’E | * | 81 |  | no | **T05** | *P. teleius* I | JX311272 | This paper |
| SR081.14 | *P. teleius* *teleius* | Ukraine, Transcarpathia, Mukachero District | 48°26’N | 22°43’E | * | 81 |  | no | **T04** | *P. teleius* I | JX311273 | This paper |
| SR087.01 | *P. teleius euphemia* | Russia, Chabarovsk, near Voronezhskoe | 48°28’N | 135°05’E | * | 87 |  | no | **T05** | *P. teleius* I | JX311275 | This paper |
| SR091.03 | *P. teleius* *sinalcon* | China, Shaanxi, Qinling mountains, Taibai Shan | 34°02’N | 107°18’E |  | 91 |  | no | **T01** | *P. teleius* IV | JX311279 | This paper |
| ZF-LY-000370 | *P. teleius ogumae* | Japan, Hokkaido, Nakasatsunai-mura, Kasai-gun, Moto-sarabetsu | 42°36’N | 143°06’E | * | 15 |  | no | **T55** | *P. teleius* IV | JX311300 | This paper |
| ZF-LY-000371 | *P. teleius ogumae* | Japan, Hokkaido, Nakasatsunai-mura, Kasai-gun, Moto-sarabetsu | 42°36’N | 143°06’E | * | 15 |  | no | **T02** | *P. teleius* I | JX311301 | This paper |
| ZF-LY-000373 | *P. teleius ogumae* | Japan, Hokkaido, Nakasatsunai-mura, Kasai-gun, Moto-sarabetsu | 42°36’N | 143°06’E | * | 15 |  | no | **T02** | *P. teleius* I | JX311302 | This paper |
| ZF-LY-000374 | *P. teleius ogumae* | Japan, Hokkaido, Nakasatsunai-mura, Kasai-gun, Moto-sarabetsu | 42°36’N | 143°06’E | * | 15 |  | no | **T02** | *P. teleius* I | JX311303 | This paper |
| ZF-LY-000378 | *P. teleius ogumae* | Japan, Hokkaido, Nakasatsunai-mura, Kasai-gun, Moto-sarabetsu | 42°36’N | 143°06’E | * | 15 |  | no | **T55** | *P. teleius* IV | JX311304 | This paper |
| ZF-LY-000384 | *P. teleius ogumae* | Japan, Hokkaido, Nakasatsunai-mura, Kasai-gun, Moto-sarabetsu | 42°36’N | 143°06’E | * | 15 |  | no | **T02** | *P. teleius* I | JX311305 | This paper |
| ZF-LY-000386 | *P. teleius ogumae* | Japan, Hokkaido, Nakasatsunai-mura, Kasai-gun, Moto-sarabetsu | 42°36’N | 143°06’E | * | 15 |  | no | **T02** | *P. teleius* I | JX311306 | This paper |
| ZF-LY-000387 | *P. teleius ogumae* | Japan, Hokkaido, Nakasatsunai-mura, Kasai-gun, Moto-sarabetsu | 42°36’N | 143°06’E | * | 15 |  | no | **T54** | *P. teleius* IV | JX311307 | This paper |
| ZF-LY-000343 | *P. teleius daisensis* | Japan, Honshu, Shimane pref., Okuizumo-cho, Nita-gun, Oh Pass | 35°09’N | 133°02’E |  | 11 |  | no | **T03** | *P. teleius* II | JX311290 | This paper |
| ZF-LY-000344 | *P. teleius daisensis* | Japan, Honshu, Shimane pref., Okuizumo-cho, Nita-gun, Oh Pass | 35°09’N | 133°02’E |  | 11 |  | no | **T03** | *P. teleius* II | JX311291 | This paper |
| ZF-LY-000345 | *P. teleius daisensis* | Japan, Kyushu, Oita pref., Kokonoe-machi, Kusu-gun, Jizohara | 33°10’N | 131°11’E |  | 12 |  | no | **T43** | *P. teleius* III | JX311292 | This paper |
| ZF-LY-000346 | *P. teleius daisensis* | Japan, Kyushu, Oita pref., Kokonoe-machi, Kusu-gun, Jizohara | 33°10’N | 131°11’E |  | 12 |  | no | **T59** | *P. teleius* III | JX311293 | This paper |
| ZF-LY-000347 | *P. teleius daisensis* | Japan, Kyushu, Oita pref., Kokonoe-machi, Kusu-gun, Jizohara | 33°10’N | 131°11’E |  | 12 |  | no | **T43** | *P. teleius* III | JX311294 | This paper |
| ZF-LY-000349 | *P. teleius daisensis* | Japan, Kyushu, Oita pref., Kokonoe-machi, Kusu-gun, Jizohara | 33°10’N | 131°11’E |  | 12 |  | no | **T59** | *P. teleius* III | JX311295 | This paper |
| ZF-LY-000354 | *P. teleius kazamoto* | Japan, Honshu, Yamanashi pref., Fujiyoshida City, Kitafuji Square | 35°26’N | 138°47’E |  | 13 |  | no | **T64** | *P. teleius* II | JX311296 | This paper |
| ZF-LY-000364 | *P. teleius daisensis* | Japan, Honshu, Hiroshima pref., Shobara City, Takano-cho, Kenashiyama Farm | 35°03’N | 132°56’E |  | 14 |  | no | **T56** | *P. teleius* II | JX311297 | This paper |
| ZF-LY-000366 | *P. teleius daisensis* | Japan, Honshu, Hiroshima pref., Shobara City, Takano-cho, Kenashiyama Farm | 35°03’N | 132°56’E |  | 14 |  | no | **T70** | *P. teleius* II | JX311298 | This paper |
| ZF-LY-000367 | *P. teleius daisensis* | Japan, Honshu, Hiroshima prefecture, Yamagata-gun, Kitahiroshima-cho, Mt. Ungetsu | 34°41’N | 132°32’E | * | 16 |  | no | **T58** | *P. teleius* II | JX311299 | This paper |
| ZF-LY-000339 | *P. teleius kazamoto* | Japan, Honshu, Yamanashi pref., Shimohagihara, Koshu City, Enzan | 35°42’N | 138°45’E |  | 7 |  | no | **T60** | *P. teleius* II | JX311286 | This paper |
| ZF-LY-000340 | *P. teleius kazamoto* | Japan, Honshu, Yamanashi pref., Shimohagihara, Koshu City, Enzan | 35°42’N | 138°45’E |  | 7 |  | no | **T49** | *P. teleius* II | JX311287 | This paper |
| ZF-LY-000341 | *P. teleius kazamoto* | Japan, Honshu, Yamanashi pref., Shimohagihara, Koshu City, Enzan | 35°42’N | 138°45’E |  | 7 |  | no | **T49** | *P. teleius* II | JX311288 | This paper |
| ZF-LY-000342 | *P. teleius kazamoto* | Japan, Honshu, Yamanashi pref., Shimohagihara, Koshu City, Enzan | 35°42’N | 138°45’E |  | 7 |  | no | **T49** | *P. teleius* II | JX311289 | This paper |
| ZF-LY-000336 | *P. teleius kazamoto* | Japan, Honshu, Nagano pref., Matsumoto, Yoriaido, Nagawa | 36°03’N | 137°41’E |  | 5 |  | no | **T57** | *P. teleius* IV | JX311285 | This paper |
| ZF-LY-000316 | *P. teleius hosonoi* | Japan, Honshu, Gifu pref., Ono-gun, Mt. Ohchozan | 36°10’N | 136°51’E |  | 2 |  | no | **T44** | *P. teleius* IV | JX311282 | This paper |
| ZF-LY-000317 | *P. teleius hosonoi* | Japan, Honshu, Gifu pref., Yoshinodani-mura, Hakusan | 36°27’N | 136°39’E |  | 3 |  | no | **T44** | *P. teleius* IV | JX311283 | This paper |
| ZF-LY-000333 | *P. teleius ogumae* | Japan, Honshu, Aomori pref., Ohma-machi, Shimokita-gun, Okoppe | 41°29’N | 140°54’E |  | 4 |  | no | **T55** | *P. teleius* IV | JX311284 | This paper |
| MG02N009 | *P. teleius* *teleius* ^$^ | Romania, Transylvania, Cluj-Napoca | 46°46’N | 23°35’E | * | 93 |  | ? | **T07** | *P. teleius* I | AY675418, HQ918140 |  |
| TDA99Q975 | *P. teleius* *teleius* ^$^ | Poland, Domaszowice | 50°52’N | 20°40’E | * | 94 |  | ? | **T67** | *P. teleius* I | AY675428, HQ918139 |  |
| TDA99Q976 | *P. teleius* *teleius* ^$^ | Poland, Krakow | 50°03’N | 19°56’E | * | 95 |  | ? | **T69** | *P. teleius* I | AY675429 |  |
| UK99W801 | *P. teleius kazamoto* ^$^ | Japan, Honshu, Yamanashi Pref., Nirasaki | 35°42’N | 138°27’E | * | 96 |  | ? | **T68** | *P. teleius* II | AY675437, HQ918138 |  |
| UK99W809 | *P. teleius kazamoto* ^$^ | Japan, Honshu, Yamanashi Pref., Kuromori | 35°54’N | 138°32’E | * | 97 |  | ? | **T66** | *P. teleius* II | AY675440, HQ918035 |  |
| TERU5 | *P. teleius* *teleius* ^$^ | Russia |  |  |  | 98 |  | ? | **T07** | *P. teleius* I | HQ918159 |  |
| RV07E460 | *P. teleius* *teleius* ^$^ | Romania, Transylvania, Brasov, Dumbrava Vandului | 45°47'N | 25°07'E | * | 99 |  | ? | **T07** | *P. teleius* I | HQ918167 |  |
| UK08J627 | *P. teleius* *euphemia* ^$^ | Mongolia, Ulaanbaatar, Bogt Uul | 47°48’N | 106°59’E | * | 120 |  | ? | **T30** | *P. teleius* Wolbachia | HQ918161 |  |
| TEJA1 | *P. teleius daisensis* ^$^ | Japan, Honshu, Hiroshima prefecture, Candelo | 34°N | 132°E | * | 121 |  | ? | **T71** | *P. teleius* II | HQ918158 |  |
| TESL6 | *P. teleius* *teleius* ^$^ | Slovakia, Trencin | 48°54’N | 18°02’E | * | 122 |  | ? | **T72** | *P. teleius* I | HQ918160 |  |
| RV-06-M891 | *P. teleius* *teleius* ^$^ | Romania, Transylvania, Bihor | 46°56'N | 22°32'E |  | n.i. |  | ? | **T04** | *P. teleius* I | HQ004727 |  |
| RV-06-M964 | *P. teleius* *teleius* ^$^ | Romania, Transylvania, Brasov, Racos | 46°02'N | 25°22'E |  | n.i. |  | ? | **T04** | *P. teleius* I | HQ004729 |  |
| RVcoll.07-C168 | *P. teleius* *teleius* ^$^ | Romania, Transylvania, Cluj-Napoca | 46°50'N | 23°38'E |  | n.i. |  | ? | **T04** | *P. teleius* I | HQ004724 |  |
| RVcoll.07-C163 | *P. teleius* *teleius* ^$^ | Romania, Transylvania, Cluj-Napoca | 46°50'N | 23°38'E |  | n.i. |  | ? | **T04** | *P. teleius* I | HQ004725 |  |
| RV-07-C162 | *P. teleius* *teleius* ^$^ | Romania, Transylvania, Cluj-Napoca | 46°50'N | 23°38'E |  | n.i. |  | ? | **T04** | *P. teleius* I | HQ004726 |  |
| RVcoll.06-M885 | *P. teleius* *teleius* ^$^ | Romania, Transylvania, Bogomaia | 46°50'N | 23°39'E |  | n.i. |  | ? | **T04** | *P. teleius* I | HQ004723 |  |
| BC ZSM Lep 21847 | *P. teleius* *teleius* ^$^ | Germany, Bavaria, Diessen | 47°56'N | 11°05'E |  | n.i. |  | ? | **T23** | *P. teleius* I | JF415712 |  |
| BC ZSM Lep 28464 | *P. teleius* *teleius* ^$^ | Germany, Bavaria, Saulburg | 48°59'N | 12°32'E |  | n.i. |  | ? | **T23** | *P. teleius* I | HM391889 |  |
| BC ZSM Lep 30669 | *P. teleius* *teleius* ^$^ | Germany, Bavaria, Passau, near Egling |  |  |  | n.i. |  | ? | **T23** | *P. teleius* I | HQ957211 |  |
| MT-Nagano1 | *P. teleius kazamoto* ^$^ | Japan, Nagano Pref., Chino City |  |  |  | n.i. |  | ? | **T66** | *P. teleius* II | AB457755 | Yago et al. (unpubl.) |
| SR019.11 | *P. nausithous* | Russia, Novosibirsk, near Akademgorodok | 54°49’N | 83°06’E |  | 19 |  | no | **N22** | *P. nausithous* I | JX311068 | This paper |
| SR020.02 | *P. nausithous* | Russia, Novosibirsk, near Akademgorodok | 54°49’N | 83°07’E |  | 20 |  | no | **N34** | *P. nausithous* I | JX311070 | This paper |
| SR020.03 | *P. nausithous* | Russia, Novosibirsk, near Akademgorodok | 54°49’N | 83°07’E |  | 20 |  | no | **N21** | *P. nausithous* I | JX311071 | This paper |
| SR021.01 | *P. nausithous* | Russia, Novosibirsk, near Akademgorodok | 54°50’N | 83°08’E |  | 21 |  | no | **N13** | *P. nausithous* I | JX311072 | This paper |
| SR021.03 | *P. nausithous* | Russia, Novosibirsk, near Akademgorodok | 54°50’N | 83°08’E |  | 21 |  | no | **N13** | *P. nausithous* I | JX311073 | This paper |
| SR021.38 | *P. nausithous* | Russia, Novosibirsk, near Akademgorodok | 54°50’N | 83°08’E |  | 21 |  | no | **N13** | *P. nausithous* I | JX311074 | This paper |
| SR022.01 | *P. nausithous* | Russia, near Barnaul | 53°22’N | 84°03’E |  | 22 |  | no | **N13** | *P. nausithous* I | JX311076 | This paper |
| SR022.03 | *P. nausithous* | Russia, near Barnaul | 53°22’N | 84°03’E |  | 22 |  | no | **N13** | *P. nausithous* I | JX311077 | This paper |
| SR022.05 | *P. nausithous* | Russia, near Barnaul | 53°22’N | 84°03’E |  | 22 |  | no | **N13** | *P. nausithous* I | JX311079 | This paper |
| SR022.06 | *P. nausithous* | Russia, near Barnaul | 53°22’N | 84°03’E |  | 22 |  | no | **N20** | *P. nausithous* I | JX311080 | This paper |
| SR022.07 | *P. nausithous* | Russia, near Barnaul | 53°22’N | 84°03’E |  | 22 |  | no | **N13** | *P. nausithous* I | JX311081 | This paper |
| SR023.03 | *P. nausithous* | Russia, Altai mountains, around Cherga | 51°33’N | 85°33’E |  | 23 |  | no | **N39** | *P. nausithous* I | JX311082 | This paper |
| SR023.31 | *P. nausithous* | Russia, Altai mountains, around Cherga | 51°33’N | 85°33’E |  | 23 |  | no | **N19** | *P. nausithous* I | JX311085 | This paper |
| SR024.03 | *P. nausithous* | Russia, Altai mountains, Choya-District, near Levinka | 51°57’N | 86°22’E |  | 24 |  | no | **N30** | *P. nausithous* I | JX311088 | This paper |
| SR024.08 | *P. nausithous* | Russia, Altai mountains, Choya-District, near Levinka | 51°57’N | 86°22’E |  | 24 |  | no | **N18** | *P. nausithous* I | JX311089 | This paper |
| SR024.36 | *P. nausithous* | Russia, Altai mountains, Choya-District, near Levinka | 51°57’N | 86°22’E |  | 24 |  | no | **N13** | *P. nausithous* I | JX311090 | This paper |
| SR025.01 | *P. nausithous* | Russia, Altai mountains, Turochak District, near Verkh Biisk | 52°02’N | 87°04’E |  | 25 |  | no | **N32** | *P. nausithous* I | JX311091 | This paper |
| SR025.02 | *P. nausithous* | Russia, Altai mountains, Turochak District, near Verkh Biisk | 52°02’N | 87°04’E |  | 25 |  | yes | **N13** | *P. nausithous* I | JX311092 | This paper |
| SR025.07 | *P. nausithous* | Russia, Altai mountains, Turochak District, near Verkh Biisk | 52°02’N | 87°04’E |  | 25 |  | no | **N16** | *P. nausithous* I | JX311094 | This paper |
| SR025.10 | *P. nausithous* | Russia, Altai mountains, Turochak District, near Verkh Biisk | 52°02’N | 87°04’E |  | 25 |  | no | **N17** | *P. nausithous* I | JX311095 | This paper |
| SR026.05 | *P. nausithous* | Russia, Altai mountains, around Ulus-Cherga | 51°31’N | 85°27’E |  | 26 |  | no | **N16** | *P. nausithous* I | JX311097 | This paper |
| SR026.06 | *P. nausithous* | Russia, Altai mountains, around Ulus-Cherga | 51°31’N | 85°27’E |  | 26 |  | no | **N38** | *P. nausithous* I | JX311098 | This paper |
| SR026.53 | *P. nausithous* | Russia, Altai mountains, around Ulus-Cherga | 51°31’N | 85°27’E |  | 26 |  | no | **N16** | *P. nausithous* I | JX311099 | This paper |
| SR028.24 | *P. nausithous* | Russia, Altai mountains, Shebalino District, near Ilyianka | 51°20’N | 85°10’E |  | 28 |  | no | **N16** | *P. nausithous* I | JX311112 | This paper |
| SR028.32 | *P. nausithous* | Russia, Altai mountains, Shebalino District, near Ilyianka | 51°20’N | 85°10’E |  | 28 |  | no | **N16** | *P. nausithous* I | JX311115 | This paper |
| SR031.01 | *P. nausithous* | N Kazakhstan, Akmola Prov., around Kokshetau | 53°16’N | 69°24’E | * | 31 |  | no | **N36** | *P. nausithous* I | JX311123 | This paper |
| SR031.02 | *P. nausithous* | N Kazakhstan, Akmola Prov., around Kokshetau | 53°16’N | 69°24’E | * | 31 |  | no | **N36** | *P. nausithous* I | JX311124 | This paper |
| SR031.03 | *P. nausithous* | N Kazakhstan, Akmola Prov., around Kokshetau | 53°16’N | 69°24’E | * | 31 |  | yes | **N41** | *P. nausithous* Wolbachia | JX311125 | This paper |
| SR032.02 | *P. nausithous* | Germany, Westerwald, around Montabaur | 50°25’N | 07°48’E |  | 32 |  | no | **N15** | *P. nausithous* I | JX311127 | This paper |
| SR034.01 | *P. nausithous* | SE France, Rhône-Alpes, Lavours Ain | 45°48’N | 05°45’E | * | 34 |  | no | **N31** | *P. nausithous* I | JX311131 | This paper |
| SR034.02 | *P. nausithous* | SE France, Rhône-Alpes, Lavours Ain | 45°48’N | 05°45’E | * | 34 |  | no | **N14** | *P. nausithous* II | JX311132 | This paper |
| SR034.03 | *P. nausithous* | SE France, Rhône-Alpes, Lavours Ain | 45°48’N | 05°45’E | * | 34 |  | no | **N14** | *P. nausithous* II | JX311133 | This paper |
| SR039.01 | *P. nausithous* | E France, Lorraine, Mortagne, Rambervilles | 48°20’N | 06°37’E | * | 39 |  | no | **N37** | *P. nausithous* I | JX311140 | This paper |
| SR039.02 | *P. nausithous* | E France, Lorraine, Mortagne, Rambervilles | 48°20’N | 06°37’E | * | 39 |  | no | **N02** | *P. nausithous* I | JX311141 | This paper |
| SR040.01 | *P. nausithous* | E France, Lorraine, Moselotte, Saint-Amé | 47°59’N | 06°45’E | * | 40 |  | no | **N26** | *P. nausithous* I | JX311142 | This paper |
| SR041.01 | *P. nausithous* | E Germany, Chemnitz, Heinersdorfer Teiche | 50°52’N | 12°53’E | * | 41 |  | no | **N03** | *P. nausithous* II | JX311143 | This paper |
| SR041.03 | *P. nausithous* | E Germany, Chemnitz, Heinersdorfer Teiche | 50°52’N | 12°53’E | * | 41 |  | no | **N03** | *P. nausithous* II | JX311144 | This paper |
| SR042.01 | *P. nausithous* | E Germany, Saxony, near Schkeuditz | 51°23’N | 12°13’E |  | 42 |  | no | **N03** | *P. nausithous* II | JX311145 | This paper |
| SR042.02 | *P. nausithous* | E Germany, Saxony, near Schkeuditz | 51°23’N | 12°13’E |  | 42 |  | no | **N03** | *P. nausithous* II | JX311146 | This paper |
| SR042.03 | *P. nausithous* | E Germany, Saxony, near Schkeuditz | 51°23’N | 12°13’E |  | 42 |  | no | **N03** | *P. nausithous* II | JX311147 | This paper |
| SR043.01 | *P. nausithous* | SW Germany, Palatinate, near Landau | 49°12’N | 08°05’E |  | 43 |  | no | **N02** | *P. nausithous* I | JX311148 | This paper |
| SR043.02 | *P. nausithous* | SW Germany, Palatinate, near Landau | 49°12’N | 08°05’E |  | 43 |  | no | **N02** | *P. nausithous* I | JX311149 | This paper |
| SR044.01 | *P. nausithous* | SW Germany, Palatinate, Queichhambach | 49°13’N | 07°58’E |  | 44 |  | no | **N02** | *P. nausithous* I | JX311150 | This paper |
| SR044.02 | *P. nausithous* | SW Germany, Palatinate, Queichhambach | 49°13’N | 07°58’E |  | 44 |  | no | **N02** | *P. nausithous* I | JX311151 | This paper |
| SR045.13 | *P. nausithous* | SW Germany, Palatinate, near Zeiskam | 49°13’N | 08°14’E |  | 45 |  | no | **N02** | *P. nausithous* I | JX311152 | This paper |
| SR047.08 | *P. nausithous* | Russia, S Ural mountains, Cheljabinsk region, near Snezhinsk | 56°05’N | 60°44’E | * | 47 |  | no | **N13** | *P. nausithous* I | JX311153 | This paper |
| SR047.11 | *P. nausithous* | Russia, S Ural mountains, Cheljabinsk region, near Snezhinsk | 56°05’N | 60°44’E | * | 47 |  | no | **N13** | *P. nausithous* I | JX311154 | This paper |
| SR047.17 | *P. nausithous* | Russia, S Ural mountains, Cheljabinsk region, near Snezhinsk | 56°05’N | 60°44’E | * | 47 |  | no | **N35** | *P. nausithous* I | JX311155 | This paper |
| SR047.18 | *P. nausithous* | Russia, S Ural mountains, Cheljabinsk region, near Snezhinsk | 56°05’N | 60°44’E | * | 47 |  | no | **N13** | *P. nausithous* I | JX311156 | This paper |
| SR048.01 | *P. nausithous* ^§^ | Russia, Volgograd region, near Vodnyi village | 48°43’N | 44°30’E | * | 48 |  | no | **N12** | *P. nausithous* I | JX311158 | This paper |
| SR048.02 | *P. nausithous* ^§^ | Russia, Volgograd region, near Vodnyi village | 48°43’N | 44°30’E | * | 48 |  | no | **N12** | *P. nausithous* I | JX311159 | This paper |
| SR048.06 | *P. nausithous* ^§^ | Russia, Volgograd region, near Vodnyi village | 48°43’N | 44°30’E | * | 48 |  | no | **N12** | *P. nausithous* I | JX311160 | This paper |
| SR048.09 | *P. nausithous* ^§^ | Russia, Volgograd region, near Vodnyi village | 48°43’N | 44°30’E | * | 48 | ♀ | yes | **N11** | *P. nausithous* Wolbachia | JX311161 | This paper |
| SR052.01 | *P. nausithous* ^§^ | Russia, Volgograd region, near Vodnyi village | 48°43’N | 44°30’E | * | 52 |  | no | **N11** | *P. nausithous* Wolbachia | JX311166 | This paper |
| SR053.08 | *P. nausithous* | Russia, S Ural mountains, Cheljabinsk region, Itkul lake | 56°21’N | 62°11’E | * | 53 |  | no | **N13** | *P. nausithous* I | JX311170 | This paper |
| SR054.01 | *P. nausithous* ^§^ | Russia, Volgograd region, Kalach-na-Dour-district, Ryumino village | 48°43’N | 43°37’E |  | 54 | ♀ | yes | **N11** | *P. nausithous* Wolbachia | JX311171 | This paper |
| SR055.01 | *P. nausithous* | E Germany, south of Kahla, Elsterwerda, Schwarze Elster | 51°28’N | 13°33’E | * | 55 |  | no | **N03** | *P. nausithous* II | JX311172 | This paper |
| SR055.02 | *P. nausithous* | E Germany, south of Kahla, Elsterwerda, Schwarze Elster | 51°28’N | 13°33’E | * | 55 |  | no | **N03** | *P. nausithous* II | JX311173 | This paper |
| SR055.03 | *P. nausithous* | E Germany, south of Kahla, Elsterwerda, Schwarze Elster | 51°28’N | 13°33’E | * | 55 |  | no | **N03** | *P. nausithous* II | JX311174 | This paper |
| SR055.04 | *P. nausithous* | E Germany, south of Kahla, Elsterwerda, Schwarze Elster | 51°28’N | 13°33’E | * | 55 |  | no | **N03** | *P. nausithous* II | JX311175 | This paper |
| SR055.05 | *P. nausithous* | E Germany, south of Kahla, Elsterwerda, Schwarze Elster | 51°28’N | 13°33’E | * | 55 |  | no | **N03** | *P. nausithous* II | JX311176 | This paper |
| SR058.01 | *P. nausithous* | Bulgaria, near Sofia, Mt. Lyulin | 42°39’N | 23°05’E | * | 58 |  | no | **N25** | *P. nausithous* I | JX311177 | This paper |
| SR058.02 | *P. nausithous* | Bulgaria, near Sofia, Mt. Lyulin | 42°39’N | 23°05’E | * | 58 |  | no | **N25** | *P. nausithous* I | JX311178 | This paper |
| SR059.01 | *P. nausithous* | Spain, Soria province, near Abejar | 41°48’N | 2°47’W |  | 59 |  | no | **N10** | *P. nausithous* II | JX311179 | This paper |
| SR060.02 | *P. nausithous* | Spain, Madrid Province, Oteruelo del Valle | 40°55’N | 3°51’W |  | 60 |  | no | **N10** | *P. nausithous* II | JX311180 | This paper |
| SR060.05 | *P. nausithous* | Spain, Madrid Province, Oteruelo del Valle | 40°55’N | 3°51’W |  | 60 |  | no | **N10** | *P. nausithous* II | JX311181 | This paper |
| SR062.05 | *P. nausithous* | E Germany, Saxony, near Schkeuditz | 51°23’N | 12°11’E |  | 62 |  | no | **N03** | *P. nausithous* II | JX311184 | This paper |
| SR062.06 | *P. nausithous* | E Germany, Saxony, near Schkeuditz | 51°23’N | 12°11’E |  | 62 |  | no | **N03** | *P. nausithous* II | JX311185 | This paper |
| SR062.07 | *P. nausithous* | E Germany, Saxony, near Schkeuditz | 51°23’N | 12°11’E |  | 62 |  | no | **N03** | *P. nausithous* II | JX311186 | This paper |
| SR062.08 | *P. nausithous* | E Germany, Saxony, near Schkeuditz | 51°23’N | 12°11’E |  | 62 |  | no | **N03** | *P. nausithous* II | JX311187 | This paper |
| SR062.09 | *P. nausithous* | E Germany, Saxony, near Schkeuditz | 51°23’N | 12°11’E |  | 62 |  | no | **N03** | *P. nausithous* II | JX311188 | This paper |
| SR064.04 | *P. nausithous* | S Germany, Lake Constance, near Constance | 47°39’N | 09°10’E | * | 64 |  | no | **N09** | *P. nausithous* II | JX311191 | This paper |
| SR064.05 | *P. nausithous* | S Germany, Lake Constance, near Constance | 47°39’N | 09°10’E | * | 64 |  | no | **N09** | *P. nausithous* II | JX311192 | This paper |
| SR066.11 | *P. nausithous* | E Poland, Kosyn | 51°23’N | 23°34’E |  | 66 |  | no | **N33** | *P. nausithous* II | JX311203 | This paper |
| SR066.12 | *P. nausithous* | E Poland, Kosyn | 51°23’N | 23°34’E |  | 66 |  | no | **N24** | *P. nausithous* I | JX311204 | This paper |
| SR066.13 | *P. nausithous* | E Poland, Kosyn | 51°23’N | 23°34’E |  | 66 |  | no | **N24** | *P. nausithous* I | JX311205 | This paper |
| SR066.14 | *P. nausithous* | E Poland, Kosyn | 51°23’N | 23°34’E |  | 66 |  | no | **N24** | *P. nausithous* I | JX311206 | This paper |
| SR067.08 | *P. nausithous* | S Poland, Wiesiółka | 50°25’N | 19°21’E |  | 67 |  | no | **N40** | *P. nausithous* II | JX311212 | This paper |
| SR068.11 | *P. nausithous* | SE Poland, Widacz | 49°38’N | 21°50’E |  | 68 |  | no | **N08** | *P. nausithous* I | JX311218 | This paper |
| SR068.13 | *P. nausithous* | SE Poland, Widacz | 49°38’N | 21°50’E |  | 68 |  | no | **N06** | *P. nausithous* I | JX311219 | This paper |
| SR068.14 | *P. nausithous* | SE Poland, Widacz | 49°38’N | 21°50’E |  | 68 |  | no | **N07** | *P. nausithous* I | JX311220 | This paper |
| SR068.15 | *P. nausithous* | SE Poland, Widacz | 49°38’N | 21°50’E |  | 68 |  | no | **N06** | *P. nausithous* I | JX311221 | This paper |
| SR069.01 | *P. nausithous* | SE Germany, Traunstein, Kirchanschöring | 47°56’N | 12°56’E | * | 69 |  | no | **N42** | *P. nausithous* I | JX311222 | This paper |
| SR070.05 | *P. nausithous* | Croatia, Bedekovičeve Grabe | 46°26’N | 16°24’E | * | 70 |  | no | **N28** | *P. nausithous* I | JX311224 | This paper |
| SR070.14 | *P. nausithous* | Croatia, Bedekovičeve Grabe | 46°26’N | 16°24’E | * | 70 |  | no | **N05** | *P. nausithous* I | JX311228 | This paper |
| SR072.01 | *P. nausithous* | E Germany, Saxony, Leipzig/Wachau | 51°16’N | 12°26’E |  | 72 |  | no | **N04** | *P. nausithous* II | JX311233 | This paper |
| SR072.02 | *P. nausithous* | E Germany, Saxony, Leipzig/Wachau | 51°16’N | 12°26’E |  | 72 |  | no | **N04** | *P. nausithous* II | JX311234 | This paper |
| SR073.01 | *P. nausithous* | E Germany, Thuringia, Jena | 50°55’N | 11°35’E |  | 73 |  | no | **N03** | *P. nausithous* II | JX311235 | This paper |
| SR073.02 | *P. nausithous* | E Germany, Thuringia, Jena | 50°55’N | 11°35’E |  | 73 |  | no | **N03** | *P. nausithous* II | JX311236 | This paper |
| SR073.03 | *P. nausithous* | E Germany, Thuringia, Jena | 50°55’N | 11°35’E |  | 73 |  | no | **N23** | *P. nausithous* II | JX311237 | This paper |
| SR073.04 | *P. nausithous* | E Germany, Thuringia, Jena | 50°55’N | 11°35’E |  | 73 |  | no | **N03** | *P. nausithous* II | JX311238 | This paper |
| SR073.05 | *P. nausithous* | E Germany, Thuringia, Jena | 50°55’N | 11°35’E |  | 73 |  | no | **N23** | *P. nausithous* II | JX311239 | This paper |
| SR073.06 | *P. nausithous* | E Germany, Thuringia, Jena | 50°55’N | 11°35’E |  | 73 |  | no | **N03** | *P. nausithous* II | JX311240 | This paper |
| SR074.02 | *P. nausithous* | SW Germany, Palatinate, Neuburg/Rhein | 48°59’N | 08°15’E |  | 74 |  | no | **N02** | *P. nausithous* I | JX311241 | This paper |
| SR074.03 | *P. nausithous* | SW Germany, Palatinate, Neuburg/Rhein | 48°59’N | 08°15’E |  | 74 |  | no | **N02** | *P. nausithous* I | JX311242 | This paper |
| SR074.04 | *P. nausithous* | SW Germany, Palatinate, Neuburg/Rhein | 48°59’N | 08°15’E |  | 74 |  | no | **N02** | *P. nausithous* I | JX311243 | This paper |
| SR074.05 | *P. nausithous* | SW Germany, Palatinate, Neuburg/Rhein | 48°59’N | 08°15’E |  | 74 |  | no | **N02** | *P. nausithous* I | JX311244 | This paper |
| SR074.07 | *P. nausithous* | SW Germany, Palatinate, Neuburg/Rhein | 48°59’N | 08°15’E |  | 74 |  | no | **N02** | *P. nausithous* I | JX311245 | This paper |
| SR074.08 | *P. nausithous* | SW Germany, Palatinate, Neuburg/Rhein | 48°59’N | 08°15’E |  | 74 |  | no | **N02** | *P. nausithous* I | JX311246 | This paper |
| SR074.09 | *P. nausithous* | SW Germany, Palatinate, Neuburg/Rhein | 48°59’N | 08°15’E |  | 74 |  | no | **N02** | *P. nausithous* I | JX311247 | This paper |
| SR077.05 | *P. nausithous* | Belarus | 51°48’N | 30°15’E | * | 77 |  | no | **N27** | *P. nausithous* I | JX311252 | This paper |
| SR077.06 | *P. nausithous* | Belarus | 51°48’N | 30°15’E | * | 77 |  | no | **N46** | *P. nausithous* I | JX311253 | This paper |
| SR078.04 | *P. nausithous* | Slovenia, Celje | 46°14’N | 15°15’E | * | 78 |  | no | **N05** | *P. nausithous* I | JX311256 | This paper |
| SR078.06 | *P. nausithous* | Slovenia, Celje | 46°14’N | 15°15’E | * | 78 |  | no | **N29** | *P. nausithous* I | JX311257 | This paper |
| SR078.07 | *P. nausithous* | Slovenia, Celje | 46°14’N | 15°15’E | * | 78 |  | no | **N05** | *P. nausithous* I | JX311258 | This paper |
| SR078.08 | *P. nausithous* | Slovenia, Celje | 46°14’N | 15°15’E | * | 78 |  | no | **N05** | *P. nausithous* I | JX311259 | This paper |
| SR083.01 | *P. nausithous* ^§^ | Romania, Transylvania, Cluj-Napoca | 46°54’N | 23°46’E |  | 83 |  | no | **N44** | *P. nausithous* I | JX311274 | This paper |
| SR088.01 | *P. nausithous* | Slovakia, Abrod | 48°42’N | 17°10’E | * | 88 | ♀ | yes | **N43** | *P. nausithous* Wolbachia | JX311276 | This paper |
| SR088.02 | *P. nausithous* | Slovakia, Abrod | 48°42’N | 17°10’E | * | 88 |  | no | **N43** | *P. nausithous* Wolbachia | JX311277 | This paper |
| SR089.01 | *P. nausithous* | Slovakia, Stará Turá, Drgonova dolina | 48°46’N | 17°40’E | * | 89 |  | no | **N43** | *P. nausithous* Wolbachia | JX311278 | This paper |
| SR101.01 | *P. nausithous* | Czech Republic, Straznice | 48°54’N | 17°19’E |  | 1 |  | no | **N01** | *P. nausithous* Wolbachia | JX311280 | This paper |
| SR101.02 | *P. nausithous* | Czech Republic, Straznice | 48°54’N | 17°19’E |  | 1 | ♀ | yes | **N01** | *P. nausithous* Wolbachia | JX311281 | This paper |
| AD00P068 | *P. nausithous* | Russia, Tula | 54°12’N | 37°37’E | * | 90 |  | ? | **N47** | *P. nausithous* I | AY675403, HQ918134 |  |
| TDA99Q966 | *P. nausithous* | SW Poland, Swidnica | 50°50’N | 16°28’E | * | 128 |  | ? | **N45** | *P. nausithous* II | AY675427, HQ918033 |  |
| ZD99S301 | *P. nausithous* | Slovakia, Abrod | 48°42’N | 17°10’E | * | 92 |  | ? | **N01** | *P. nausithous* Wolbachia | AY675446, HQ918135 |  |
| NACZ1 | *P. nausithous* | Czech Republic, Mecichov | 49°21’N | 13°49’E | * | 123 |  | ? | **N02** | *P. nausithous* I | HQ918151 |  |
| RV08L884 | *P. nausithous* | Spain, Soria province, near Abejar | 41°48’N | 2°47’W | * | 124 |  | ? | **N10** | *P. nausithous* II | HQ918150 |  |
| NAGE2 | *P. nausithous* | Germany, Bavaria |  |  |  | 125 |  | ? | **N13** | *P. nausithous* I | HQ918152 |  |
| RV07C331 | *P. nausithous* | Romania, Moldavia, Suceava, Radauti | 47°47'N | 25°54'E | * | 126 |  | ? | **N48** | *P. nausithous* I | HQ918166 |  |
| RV06M889 | *P. nausithous* | Romania, Transylvania, Cluj-Napoca | 46°50'N | 23°40'E | * | 127 |  | ? | **N49** | *P. nausithous* I | HQ918165 |  |
| RVcoll.07-C334 | *P. nausithous* | Romania, Moldavia, Suceava, Radauti | 47°47'N | 25°54'E |  | n.i. |  | ? | **N13** | *P. nausithous* I | HQ004715 |  |
| RV-07-C116 | *P. nausithous* | Romania, Transylvania, Cluj-Napoca | 46°50'N | 23°38'E |  | n.i. |  | ? | **N13** | *P. nausithous* I | HQ004720 |  |
| RVcoll.07-C164 | *P. nausithous* | Romania, Transylvania, Cluj-Napoca | 46°50'N | 23°39'E |  | n.i. |  | ? | **N13** | *P. nausithous* I | HQ004717 |  |
| RVcoll.06-M917 | *P. nausithous* | Romania, Transylvania, Cluj-Napoca | 46°50'N | 23°39'E |  | n.i. |  | ? | **N13** | *P. nausithous* I | HQ004718 |  |
| RVcoll.07-C167 | *P. nausithous* | Romania, Transylvania, Cluj-Napoca | 46°50'N | 23°38'E |  | n.i. |  | ? | **N13** | *P. nausithous* I | GU675622 | IBoL unpubl. |
| RVcoll.07-C161 | *P. nausithous* | Romania, Transylvania, Cluj-Napoca | 46°50'N | 23°38'E |  | n.i. |  | ? | **N13** | *P. nausithous* I | GU675623 |  |
| RVcoll.06-M890 | *P. nausithous* | Romania, Transylvania, Cluj-Napoca | 46°50'N | 23°39'E |  | n.i. |  | ? | **N49** | *P. nausithous* I | GU675624 |  |
| RVcoll.07-C165 | *P. nausithous* | Romania, Transylvania, Cluj-Napoca | 46°50'N | 23°39'E |  | n.i. |  | ? | **N49** | *P. nausithous* I | HQ004716 |  |
| RV-07-C332 | *P. nausithous* | Romania, Moldavia, Suceava, Radauti | 47°47'N | 25°54'E |  | n.i. |  | ? | **N50** | *P. nausithous* I | HQ004722 |  |
| BC ZSM Lep 28463 | *P. nausithous* | Germany, Bavaria, Saulburg | 48°59'N | 12°32'E |  | n.i. |  | ? | **N51** | *P. nausithous* I | HM391888 |  |
| BC ZSM Lep 30498 | *P. nausithous* | Germany, Bavaria, Zellwies Koenigsdorf |  |  |  | n.i. |  | ? | **N52** | *P. nausithous* I | GU688438 |  |
| 2005-LOWA-542 | *P. nausithous* | Russia | 50°54'N | 106°00'E |  | n.i. |  | ? | **Chimaera^&^** |  | FJ663755 |  |
| SY03A500 | *P. albida* | China, Jiangjin |  |  |  |  |  | ? |  |  | AY675423, HQ918037 |  |
| SY03A501 | *P. atroguttata* | Taiwan, Heping |  |  |  |  |  | ? |  |  | AY675424, HQ918038 |  |
| SY03A503 | *P. daitozanus* | Taiwan, Heping |  |  |  |  |  | ? |  |  | AY675425, HQ918039 |  |
| TDA99Q985 | *P. alcon* | Denmark |  |  |  |  |  | ? |  |  | AY675431, HQ918119 |  |
| TDA99Q989 | *P. arion* | Sweden |  |  |  |  |  | ? |  |  | AY675433, HQ918130 |  |
| MW99045 | *P. arion* | Turkey, Erzurum, Köşkköy | 40°05’N | 41°26’E |  |  |  | ? |  |  | AY557034 |  |

Shading color on the left table part corresponds to subspecific membership of the species (cyan: nominate species, peach: *P. teleius obscurata*, blue: *P. t. euphemia*, orange: *P. t. sinalcon*, pink: *P. t. ogumae*, yellow: *P. t. kazamoto*, green: *P. t. hosonoi*, violet: *P. t. daisensis*) and on the right table part to COI haplogroup membership resulting from network analyses (see Fig. 2).

N Sample size

w+ *Wolbachia* infection status

? *Wolbachia* infection unknown

n.i. Barcode sequences which were not included in haplotype network construction and phylogenetic analyses due to low coverage with our sequences.

* Coordinates of these populations were inferred from locality information using Google Earth and are only approximate.

# These individuals were assigned to ssp. *obscurata* according to although the range limits of this subspecies are debated.

$ These individuals retrieved from Genebank were not assigned to a certain subspecies. Our assignment is based on .

§ These individuals were assigned as subspecies "*kijevensis*" by the specimen collector.

& This barcode sequence of *Phengaris nausithous* from Russia differs strongly (more than 6.8%) from all other *Phengaris* sequences and turned out to be a chimaera of *Phengaris teleius* (5’ half) and *Brenthis ino* (3’ half). It was therefore excluded from further analysis. Its voucher specimen is figured in BOLD (2005-LOWA-542) and actually represents *P. teleius*. Females of *P. teleius* can be very dark in Transbaikalia and are therefore easily confused with *P. nausithous* which does not seem to occur in the area. Earlier records from Transbaikalia (Amazar) were questioned by . The 3’ half of the sequence was identical to two sequences of *Brenthis ino* used in the same study (FJ663337 & FJ663338; ) and whose vouchers had been caught at the same location and on the same date as the *P. teleius* specimen.

References:

1. Als TD, Vila R, Kandul NP, Nash DR, Yen SH, et al. (2004) The evolution of alternative parasitic life histories in large blue butterflies. Nature 432: 386-390.

2. Ugelvig LV, Vila R, Pierce NE, Nash DR (2011) A phylogenetic revision of the *Glaucopsyche* section (Lepidoptera: Lycaenidae), with special focus on the *Phengaris-Maculinea* clade. Mol Phylogenet Evol 61: 237-243.

3. Dincă V, Zakharov E, Hebert PD, Vila R (2011) Complete DNA barcode reference library for a country's butterfly fauna reveals high performance for temperate Europe. P R Soc B 278: 347-355.

4. Hausmann A, Haszprunar G, Segerer AH, Speidel W, Behounek G, et al. (2011) Now DNA-barcoded: the butterflies and larger moths of Germany. Spixiana 34: 47-58.

5. Lukhtanov VA, Sourakov A, Zakharov EV, Hebert PD (2009) DNA barcoding Central Asian butterflies: increasing geographical dimension does not significantly reduce success of species identification. Mol Ecol Resources 9: 1302-1310.

6. Wiemers M, Fiedler K (2007) Does the DNA barcoding gap exist? - a case study in blue butterflies (Lepidoptera: Lycaenidae). Front Zool 4: 8.

7. Tshikolovets VV, Bidzilya A, Golovushkin M (2002) The butterflies of Transbaikal Siberia. Kyiv-Brno: Author's edition. 320 p.

8. Tshikolovets VV, Yakovlev RV, Bálint Z (2009) The butterflies of Mongolia. Kiev: Tshikolovets Publ.

9. Tshikolovets VV, Yakovlev RV, Kosterin OE (2009) The butterflies of Altai, Sayan and Tuva. Kiev: Tshikolovets Publ.

10. Sibatani A, Saigusa T, Hirowatari T (1994) The genus *Maculinea* van Eecke, 1915 (Lepidoptera: Lycaenidae) from the East Palaearctic Region. Tyô to Ga 44: 157-220.

11. Dubatolov VV, Kosterin EO (1999) Butterflies (Lepidoptera, Hesperioidea, Papilionoidea) of the Argun' basin. Nasekomye Daurii i sopredel'nykh territorii (Sbornik nauchnykh trudov) [Insects of Dauria and Adjacent Terrtories (Collected scientific works)]. Novosibirsk. pp. 195-221.
