# Supplementary material for: Wolbachia Infections Mimic Cryptic Speciation in Two Parasitic Butterfly Species, Phengaris teleius and P. nausithous (Lepidoptera: Lycaenidae)
Source: PLoS One. 2013 Nov 6;8(11):e78107. doi: 10.1371/journal.pone.0078107 (PMC3819333; doi:10.1371/journal.pone.0078107)
Supplement: Table S2 — Microsatellite genotypes of Phengaris teleius and P. nausithous . (DOC) [file pone.0078107.s005.doc]

**Table S2.** Microsatellite genotypes of *Phengaris teleius* and *Phengaris nausithous* (light grey shade) used for analysis.

| **Specimen voucher** | **1a** | **1b** | **3a** | **3b** | **7a** | **7b** | **15a** | **15b** | **8a** | **8b** | **16a** | **16b** | **9a** | **9b** | **11a** | **11b** |
| --- | --- | --- | --- | --- | --- | --- | --- | --- | --- | --- | --- | --- | --- | --- | --- | --- |
| SR006.01 | 99 | 99 | 118 | 118 | 114 | 135 | 135 | 135 |  |  | 343 | 343 | 168 | 173 | 188 | 188 |
| SR006.03 | 99 | 99 |  |  | 135 | 135 | 126 | 135 |  |  | 343 | 343 | 156 | 156 | 188 | 188 |
| SR006.05 | 99 | 99 | 112 | 112 | 114 | 135 | 126 | 135 |  |  | 335 | 337 | 156 | 156 | 188 | 188 |
| SR006.06 | 99 | 99 | 112 | 112 | 135 | 135 | 126 | 135 | 213 | 214 | 343 | 347 | 156 | 170 | 188 | 188 |
| SR006.07 | 99 | 99 |  |  | 135 | 135 | 126 | 135 |  |  | 345 | 345 | 156 | 172 | 188 | 188 |
| SR006.08 | 102 | 102 | 112 | 112 | 135 | 135 | 126 | 135 | 216 | 216 | 331 | 331 | 170 | 172 | 188 | 188 |
| SR006.10 | 117 | 128 | 110 | 124 | 114 | 114 | 131 | 139 | 186 | 186 | 273 | 353 | 156 | 156 | 188 | 188 |
| SR008.01 | 99 | 99 | 112 | 112 | 135 | 135 | 135 | 135 | 186 | 193 |  |  | 156 | 156 | 188 | 188 |
| SR009.01 |  |  |  |  |  |  |  |  | 186 | 196 | 273 | 273 | 156 | 156 | 188 | 188 |
| SR010.01 | 119 | 123 | 110 | 124 | 114 | 114 | 128 | 137 | 196 | 196 | 363 | 367 | 156 | 156 | 188 | 188 |
| SR010.14 | 111 | 125 | 110 | 124 | 114 | 114 | 137 | 137 | 196 | 196 | 337 | 339 | 156 | 156 | 188 | 188 |
| SR010.15 | 121 | 121 | 110 | 110 | 114 | 114 | 137 | 137 | 186 | 188 | 310 | 349 | 156 | 156 | 189 | 189 |
| SR010.17 | 111 | 125 | 110 | 110 | 114 | 114 | 137 | 139 | 186 | 186 | 308 | 343 | 156 | 158 | 188 | 188 |
| SR010.21 | 125 | 125 | 110 | 112 | 114 | 114 | 137 | 137 | 186 | 196 | 308 | 328 | 156 | 158 | 188 | 188 |
| SR010.32 | 123 | 134 | 110 | 110 | 114 | 114 | 128 | 137 | 186 | 193 | 315 | 320 | 156 | 156 | 188 | 188 |
| SR010.38 | 121 | 139 | 110 | 110 | 114 | 114 | 137 | 139 | 186 | 186 | 308 | 343 | 173 | 173 | 188 | 188 |
| SR010.46 | 117 | 137 | 110 | 110 | 114 | 114 | 137 | 139 | 192 | 198 | 308 | 310 | 156 | 156 | 188 | 188 |
| SR019.21 | 104 | 123 | 110 | 124 | 114 | 114 | 137 | 139 | 186 | 186 | 308 | 308 | 156 | 158 | 188 | 188 |
| SR021.44 | 121 | 134 | 110 | 110 | 114 | 114 | 137 | 141 | 186 | 190 | 339 | 343 | 156 | 156 | 188 | 188 |
| SR022.04 | 113 | 142 | 110 | 110 | 114 | 114 | 141 | 141 | 188 | 198 | 308 | 317 | 156 | 173 | 188 | 188 |
| SR023.09 | 117 | 125 | 110 | 124 | 114 | 114 | 137 | 139 | 182 | 186 | 308 | 315 | 156 | 156 | 188 | 188 |
| SR023.10 | 140 | 140 | 110 | 110 | 114 | 114 | 139 | 139 | 186 | 186 | 308 | 326 | 156 | 156 | 188 | 188 |
| SR023.33 | 142 | 152 | 110 | 124 | 114 | 114 | 137 | 137 | 186 | 196 | 359 | 361 | 156 | 156 | 188 | 188 |
| SR024.01 | 137 | 140 | 110 | 110 | 114 | 114 | 137 | 137 | 186 | 186 | 308 | 310 | 156 | 168 | 188 | 189 |
| SR025.04 | 125 | 134 | 124 | 124 | 114 | 114 | 137 | 137 | 186 | 186 | 308 | 308 | 156 | 156 | 188 | 188 |
| SR025.12 | 125 | 140 | 110 | 124 | 114 | 114 | 137 | 139 | 186 | 186 | 310 | 315 | 156 | 156 | 188 | 188 |
| SR027.01 | 123 | 132 | 110 | 124 | 114 | 114 | 137 | 137 | 186 | 188 | 308 | 308 | 156 | 156 | 188 | 188 |
| SR027.03 | 128 | 130 | 110 | 124 | 114 | 114 | 137 | 139 | 186 | 188 | 308 | 332 | 156 | 156 | 188 | 188 |
| SR027.04 | 104 | 125 | 110 | 124 | 114 | 114 | 137 | 141 | 186 | 186 | 310 | 347 | 175 | 177 | 188 | 188 |
| SR027.08 | 121 | 142 | 110 | 110 | 114 | 114 | 137 | 141 | 186 | 188 | 308 | 328 | 175 | 177 | 188 | 188 |
| SR027.12 | 121 | 132 | 110 | 124 | 114 | 114 | 137 | 137 | 186 | 186 | 308 | 343 | 156 | 156 | 188 | 188 |
| SR028.01 | 117 | 121 | 124 | 124 | 114 | 114 | 137 | 137 | 188 | 196 | 343 | 349 | 156 | 156 | 188 | 188 |
| SR028.02 | 132 | 152 | 110 | 124 | 114 | 114 | 137 | 137 | 186 | 188 | 343 | 355 | 156 | 173 | 188 | 188 |
| SR028.07 | 117 | 134 | 110 | 124 | 114 | 114 | 137 | 139 | 188 | 188 | 308 | 343 | 156 | 156 | 188 | 188 |
| SR028.15 | 130 | 134 | 110 | 112 | 114 | 114 | 137 | 141 | 188 | 196 | 308 | 345 | 156 | 173 | 188 | 188 |
| SR028.16 |  |  |  |  |  |  |  |  | 186 | 186 | 308 | 315 | 156 | 173 | 188 | 188 |
| SR028.18 | 119 | 123 | 110 | 124 | 114 | 114 | 137 | 137 | 186 | 186 | 308 | 313 | 156 | 173 | 188 | 188 |
| SR028.21 | 125 | 139 | 110 | 116 | 114 | 114 | 137 | 137 | 186 | 188 | 308 | 310 | 156 | 156 | 188 | 188 |
| SR028.26 | 111 | 134 | 124 | 124 | 114 | 114 | 137 | 139 | 186 | 188 | 308 | 339 | 156 | 156 | 188 | 188 |
| SR028.28 | 125 | 142 | 110 | 110 | 114 | 114 | 137 | 141 | 162 | 196 | 315 | 315 | 156 | 156 | 188 | 189 |
| SR029.03 | 121 | 128 | 110 | 110 | 114 | 114 | 137 | 139 | 186 | 186 | 308 | 308 | 155 | 156 | 188 | 188 |
| SR029.05 | 104 | 125 | 110 | 110 | 114 | 114 | 137 | 141 | 186 | 186 | 308 | 315 | 150 | 156 | 188 | 188 |
| SR029.07 | 123 | 128 | 110 | 124 | 114 | 114 | 137 | 137 | 186 | 186 | 349 | 353 | 150 | 150 | 188 | 189 |
| SR029.09 | 104 | 128 | 110 | 110 | 114 | 114 | 137 | 137 | 186 | 186 | 308 | 315 | 150 | 156 | 180 | 188 |
| SR029.10 | 115 | 121 | 110 | 124 | 114 | 114 | 137 | 137 | 186 | 186 | 308 | 308 |  |  |  |  |
| SR029.12 | 121 | 134 | 110 | 110 | 114 | 114 | 128 | 137 | 186 | 186 | 308 | 308 | 156 | 156 |  |  |
| SR029.13 | 127 | 132 | 110 | 124 | 114 | 114 | 128 | 137 | 186 | 188 | 308 | 353 | 156 | 156 | 188 | 188 |
| SR032.01 | 119 | 119 | 110 | 110 | 114 | 114 | 123 | 137 | 186 | 188 | 349 | 349 | 149 | 156 | 188 | 189 |
| SR032.03 | 119 | 119 | 110 | 124 | 114 | 114 | 137 | 137 | 186 | 186 | 349 | 349 | 156 | 158 | 188 | 188 |
| SR033.01 | 119 | 123 | 110 | 110 | 114 | 114 | 137 | 137 | 186 | 186 | 292 | 349 | 150 | 156 | 188 | 188 |
| SR033.02 | 119 | 119 | 110 | 124 | 114 | 114 | 128 | 139 | 186 | 192 | 339 | 347 | 150 | 156 | 184 | 188 |
| SR035.01 | 125 | 144 | 110 | 124 | 114 | 114 | 137 | 137 | 186 | 196 | 357 | 357 | 156 | 158 | 188 | 190 |
| SR036.01 | 132 | 134 | 110 | 124 | 114 | 114 | 137 | 139 | 186 | 196 | 308 | 351 | 156 | 156 | 188 | 188 |
| SR036.02 | 144 | 146 | 110 | 124 | 116 | 116 | 139 | 139 | 186 | 196 | 351 | 353 | 150 | 156 | 148 | 188 |
| SR036.04 | 134 | 134 | 110 | 110 | 114 | 114 | 137 | 137 | 186 | 196 | 308 | 357 | 150 | 156 | 148 | 148 |
| SR038.01 | 121 | 121 | 110 | 110 | 114 | 114 | 137 | 137 | 186 | 186 | 347 | 347 | 156 | 156 | 148 | 188 |
| SR038.03 | 121 | 127 | 110 | 124 | 114 | 114 | 133 | 137 | 186 | 186 | 347 | 347 | 150 | 156 | 148 | 148 |
| SR047.19 | 115 | 117 | 110 | 110 | 114 | 114 | 137 | 139 | 186 | 198 | 308 | 308 | 156 | 156 | 188 | 188 |
| SR050.01 | 119 | 123 | 110 | 124 | 114 | 114 | 137 | 137 | 186 | 186 | 345 | 347 |  |  | 188 | 188 |
| SR050.03 | 119 | 130 | 110 | 110 | 114 | 114 | 137 | 139 | 186 | 196 | 310 | 347 | 156 | 156 |  |  |
| SR050.05 | 115 | 119 | 110 | 110 | 114 | 114 | 137 | 137 | 186 | 196 | 308 | 308 | 156 | 156 |  |  |
| SR051.01 | 117 | 117 | 110 | 110 | 114 | 114 | 137 | 139 | 186 | 196 | 270 | 308 | 170 | 183 | 190 | 190 |
| SR053.01 | 119 | 119 | 110 | 124 | 114 | 114 | 137 | 141 | 186 | 186 | 313 | 349 | 183 | 183 | 190 | 190 |
| SR053.02 | 119 | 119 | 110 | 110 | 114 | 114 | 137 | 137 | 186 | 188 | 308 | 349 | 168 | 175 | 190 | 190 |
| SR053.03 | 113 | 127 | 124 | 124 | 114 | 114 | 137 | 137 | 186 | 186 | 306 | 351 | 172 | 172 | 190 | 190 |
| SR062.01 | 123 | 123 | 124 | 124 | 114 | 114 | 131 | 137 | 186 | 186 | 355 | 355 | 172 | 173 | 190 | 190 |
| SR063.01 | 115 | 123 | 110 | 124 | 114 | 114 | 137 | 137 | 186 | 196 | 351 | 351 | 172 | 183 | 190 | 190 |
| SR063.03 | 115 | 115 | 124 | 124 | 114 | 114 | 128 | 137 | 196 | 198 | 308 | 367 | 168 | 173 | 190 | 190 |
| SR065.02 | 106 | 106 | 110 | 124 | 114 | 114 | 133 | 137 | 184 | 186 | 280 | 324 | 168 | 170 | 190 | 190 |
| SR065.03 | 113 | 113 | 110 | 110 | 116 | 116 | 139 | 139 | 186 | 186 | 302 | 355 | 168 | 177 | 190 | 190 |
| SR065.04 | 119 | 121 | 110 | 110 | 114 | 114 | 137 | 137 | 186 | 186 | 302 | 308 | 170 | 172 | 190 | 190 |
| SR065.05 | 123 | 123 | 110 | 124 | 114 | 114 | 133 | 137 | 186 | 200 | 347 | 355 | 170 | 170 | 190 | 190 |
| SR065.06 | 121 | 140 | 110 | 110 | 114 | 114 | 137 | 139 | 186 | 186 | 302 | 343 | 168 | 168 | 188 | 188 |
| SR066.02 | 121 | 127 | 110 | 124 | 114 | 114 | 137 | 141 | 186 | 186 | 308 | 315 | 156 | 173 | 188 | 188 |
| SR066.03 | 125 | 128 | 110 | 110 | 114 | 114 | 137 | 137 | 186 | 186 | 345 | 349 | 156 | 172 | 188 | 188 |
| SR066.04 | 119 | 119 | 110 | 110 | 114 | 114 | 135 | 137 | 186 | 186 | 347 | 349 | 156 | 156 | 188 | 188 |
| SR066.05 | 108 | 128 | 110 | 110 | 114 | 114 | 137 | 137 | 186 | 186 | 345 | 361 | 156 | 172 | 188 | 188 |
| SR066.06 | 111 | 121 | 112 | 126 | 116 | 116 | 139 | 139 | 186 | 186 | 308 | 353 | 156 | 156 | 188 | 188 |
| SR067.02 | 104 | 119 | 124 | 124 | 114 | 114 | 137 | 137 | 186 | 196 | 343 | 361 | 156 | 168 | 188 | 188 |
| SR067.03 | 121 | 134 | 112 | 112 | 116 | 116 | 139 | 139 | 186 | 186 | 347 | 347 | 156 | 156 | 188 | 188 |
| SR067.04 | 117 | 121 | 110 | 110 | 114 | 114 | 135 | 139 |  |  |  |  | 156 | 156 | 188 | 188 |
| SR067.05 | 104 | 123 | 110 | 110 | 114 | 114 | 137 | 137 | 186 | 193 | 357 | 361 | 156 | 156 | 188 | 188 |
| SR067.06 | 117 | 117 | 110 | 110 | 114 | 114 | 137 | 137 | 186 | 193 | 347 | 353 | 156 | 156 | 188 | 188 |
| SR068.02 | 123 | 123 | 110 | 110 | 114 | 114 | 128 | 128 | 196 | 196 | 308 | 353 |  |  | 184 | 190 |
| SR068.03 | 121 | 121 | 112 | 126 | 116 | 116 | 139 | 139 | 186 | 186 | 308 | 361 | 156 | 156 | 188 | 188 |
| SR068.04 | 119 | 119 | 110 | 126 | 116 | 116 | 139 | 139 | 186 | 192 | 345 | 345 | 156 | 172 | 188 | 188 |
| SR068.05 | 119 | 125 | 110 | 124 | 114 | 114 | 137 | 141 | 186 | 186 | 351 | 355 | 156 | 156 | 188 | 188 |
| SR068.06 | 117 | 132 | 110 | 110 | 114 | 114 | 137 | 139 | 186 | 186 | 308 | 353 | 156 | 156 | 188 | 188 |
| SR070.02 | 119 | 130 | 110 | 124 | 114 | 114 | 137 | 137 | 186 | 186 | 351 | 351 | 156 | 168 | 188 | 188 |
| SR070.08 | 132 | 137 | 124 | 124 | 114 | 114 | 137 | 137 | 186 | 186 | 345 | 351 | 156 | 158 | 188 | 188 |
| SR070.09 | 104 | 119 | 124 | 124 | 114 | 114 | 137 | 137 | 186 | 186 | 345 | 353 | 168 | 168 | 188 | 188 |
| SR070.13 | 119 | 137 | 110 | 124 | 114 | 114 | 137 | 137 | 186 | 196 | 345 | 355 | 156 | 168 | 188 | 188 |
| SR070.16 | 130 | 132 | 110 | 110 | 114 | 114 | 135 | 137 | 186 | 192 | 347 | 353 | 156 | 168 | 188 | 188 |
| SR070.19 | 104 | 121 | 126 | 126 | 116 | 116 | 139 | 139 | 186 | 186 | 371 | 373 | 155 | 168 |  |  |
| SR070.28 | 111 | 119 | 110 | 110 | 114 | 114 | 137 | 137 | 186 | 186 | 308 | 347 | 168 | 168 | 188 | 188 |
| SR070.31 | 121 | 121 | 124 | 124 | 114 | 114 | 137 | 137 | 186 | 196 | 343 | 345 | 168 | 168 | 188 | 188 |
| SR075.04 | 99 | 108 | 110 | 110 | 114 | 149 | 161 | 161 |  |  | 273 | 273 |  |  |  |  |
| SR075.08 | 104 | 108 | 110 | 110 | 149 | 149 | 161 | 182 |  |  | 273 | 273 | 156 | 156 | 188 | 188 |
| SR077.02 | 119 | 134 | 124 | 124 | 114 | 114 | 139 | 139 | 186 | 196 | 308 | 351 | 156 | 156 |  |  |
| SR077.03 | 123 | 130 | 110 | 110 | 114 | 114 | 137 | 137 | 186 | 186 | 332 | 332 | 156 | 168 | 188 | 188 |
| SR078.02 | 119 | 121 | 124 | 124 | 114 | 114 | 137 | 137 | 196 | 198 | 345 | 345 | 156 | 158 | 188 | 188 |
| SR078.03 | 104 | 123 | 110 | 124 | 114 | 114 | 137 | 137 | 186 | 186 | 353 | 353 |  |  |  |  |
| SR078.10 | 123 | 128 | 110 | 110 | 114 | 114 | 137 | 141 | 186 | 198 | 343 | 355 | 156 | 168 | 188 | 188 |
| SR079.03 | 119 | 121 | 110 | 124 | 114 | 114 | 137 | 137 | 186 | 188 | 345 | 355 | 156 | 168 |  |  |
| SR079.04 | 119 | 128 | 110 | 110 | 114 | 114 | 137 | 137 | 186 | 188 | 353 | 359 | 156 | 168 | 188 | 188 |
| SR079.06 | 128 | 128 | 110 | 110 | 114 | 114 | 137 | 137 | 186 | 188 | 345 | 359 | 156 | 168 | 188 | 188 |
| SR079.07 | 111 | 132 | 126 | 126 | 116 | 116 | 139 | 139 | 188 | 196 | 345 | 345 | 168 | 168 |  |  |
| SR080.02 | 104 | 113 | 110 | 110 | 114 | 114 | 137 | 137 | 186 | 188 | 351 | 353 | 156 | 156 | 188 | 188 |
| SR080.03 | 130 | 130 | 110 | 110 | 114 | 114 | 137 | 137 | 186 | 186 | 353 | 353 | 156 | 156 |  |  |
| SR080.05 | 113 | 113 | 110 | 110 | 114 | 114 | 137 | 137 | 186 | 186 | 351 | 353 | 156 | 156 | 188 | 188 |
| SR081.02 | 104 | 121 | 124 | 124 | 114 | 114 | 137 | 137 | 186 | 186 | 326 | 339 | 156 | 156 | 188 | 188 |
| SR081.06 | 121 | 142 | 110 | 124 | 114 | 114 | 137 | 137 | 186 | 186 | 308 | 308 | 156 | 156 | 188 | 188 |
| SR081.09 | 119 | 119 | 110 | 124 | 114 | 114 | 137 | 137 | 186 | 186 | 349 | 353 | 156 | 156 | 188 | 188 |
| SR081.13 | 119 | 121 | 110 | 110 | 114 | 114 | 137 | 137 | 186 | 186 | 313 | 353 | 156 | 156 | 188 | 188 |
| SR087.01 | 130 | 130 | 110 | 110 | 114 | 114 | 137 | 154 | 186 | 186 | 273 | 273 | 156 | 162 | 188 | 190 |
| SR091.03 | 119 | 121 | 110 | 110 | 135 | 135 | 135 | 144 | 188 | 188 | 294 | 297 | 156 | 156 | 164 | 189 |
| ZF-LY-000370 | 99 | 99 | 110 | 110 | 135 | 135 | 141 | 141 | 186 | 200 | 300 | 300 | 156 | 158 | 189 | 189 |
| ZF-LY-000371 | 100 | 102 | 110 | 110 | 135 | 135 | 139 | 146 | 188 | 188 | 369 | 371 | 156 | 158 | 189 | 189 |
| ZF-LY-000373 |  |  |  |  |  |  |  |  | 200 | 201 | 306 | 373 | 158 | 158 | 189 | 189 |
| ZF-LY-000374 | 100 | 102 | 110 | 110 | 114 | 135 | 141 | 148 | 200 | 200 | 359 | 371 | 158 | 158 | 189 | 189 |
| ZF-LY-000378 | 100 | 100 | 110 | 110 | 114 | 135 | 141 | 141 | 200 | 201 | 306 | 306 | 158 | 158 | 189 | 189 |
| ZF-LY-000379 | 100 | 102 | 110 | 110 | 135 | 135 | 141 | 141 | 184 | 184 |  |  | 156 | 158 | 189 | 189 |
| ZF-LY-000384 | 100 | 100 | 110 | 110 | 135 | 135 | 141 | 141 | 186 | 200 | 359 | 359 | 158 | 158 | 189 | 189 |
| ZF-LY-000386 |  |  |  |  |  |  |  |  | 200 | 200 | 357 | 359 | 158 | 158 | 189 | 189 |
| ZF-LY-000387 | 100 | 100 | 110 | 110 | 112 | 135 | 141 | 141 | 200 | 200 | 306 | 306 | 156 | 158 | 189 | 189 |
| ZF-LY-000343 | 102 | 102 | 102 | 102 | 114 | 135 | 152 | 154 |  |  | 335 | 337 | 156 | 156 | 148 | 188 |
| ZF-LY-000344 | 102 | 102 |  |  | 114 | 114 | 152 | 158 |  |  |  |  |  |  |  |  |
| ZF-LY-000345 | 102 | 102 | 102 | 102 | 135 | 135 |  |  | 186 | 186 | 343 | 347 | 156 | 156 | 188 | 188 |
| ZF-LY-000346 | 102 | 102 |  |  | 135 | 135 | 156 | 170 |  |  |  |  | 156 | 156 | 188 | 188 |
| ZF-LY-000347 | 102 | 102 | 102 | 102 | 135 | 135 | 156 | 170 | 186 | 186 | 313 | 313 |  |  |  |  |
| ZF-LY-000348 | 99 | 99 | 124 | 124 | 135 | 135 | 172 | 172 | 165 | 216 | 339 | 339 |  |  |  |  |
| ZF-LY-000349 | 99 | 99 | 110 | 110 | 112 | 135 | 158 | 158 | 186 | 186 | 337 | 339 | 156 | 156 | 148 | 188 |
| ZF-LY-000354 | 99 | 99 | 110 | 110 | 114 | 135 | 135 | 144 | 165 | 165 | 308 | 339 | 156 | 156 | 188 | 189 |
| ZF-LY-000364 | 102 | 102 | 110 | 110 | 135 | 135 | 144 | 152 | 165 | 165 | 339 | 339 | 156 | 156 | 148 | 148 |
| ZF-LY-000366 | 102 | 102 | 110 | 110 | 135 | 135 | 154 | 154 |  |  | 328 | 328 | 156 | 156 | 188 | 188 |
| ZF-LY-000367 | 102 | 108 |  |  | 135 | 135 | 178 | 180 |  |  | 320 | 320 | 156 | 156 | 148 | 188 |
| ZF-LY-000339 | 99 | 102 | 102 | 106 | 114 | 135 | 135 | 187 | 186 | 186 | 300 | 300 | 156 | 156 | 148 | 188 |
| ZF-LY-000340 | 102 | 102 | 102 | 102 | 114 | 114 |  |  |  |  |  |  | 156 | 156 | 148 | 188 |
| ZF-LY-000341 | 102 | 102 | 104 | 104 | 114 | 114 | 187 | 187 | 186 | 186 |  |  | 156 | 156 | 148 | 184 |
| ZF-LY-000342 | 100 | 102 | 102 | 102 | 114 | 114 | 135 | 199 | 186 | 186 |  |  | 156 | 156 | 148 | 148 |
| ZF-LY-000336 | 102 | 102 | 110 | 110 | 112 | 135 | 156 | 202 | 186 | 186 | 278 | 292 | 156 | 156 | 188 | 188 |
| ZF-LY-000316 | 99 | 106 | 110 | 114 | 114 | 114 | 137 | 144 |  |  | 297 | 297 | 156 | 156 | 189 | 189 |
| ZF-LY-000317 | 100 | 104 | 110 | 110 | 114 | 114 | 137 | 152 | 190 | 190 | 302 | 302 | 156 | 156 | 174 | 184 |
| ZF-LY-000333 | 100 | 100 | 110 | 110 | 135 | 135 | 128 | 137 | 184 | 186 |  |  | 160 | 160 | 188 | 189 |
| SR019.11 | 104 | 106 | 142 | 148 | 120 | 122 | 135 | 139 | 190 | 203 | 267 | 292 | 164 | 172 | 190 | 190 |
| SR020.02 | 104 | 104 | 161 | 163 | 118 | 120 | 133 | 133 | 192 | 192 | 271 | 349 | 183 | 217 | 190 | 190 |
| SR021.01 | 104 | 104 | 145 | 150 | 120 | 120 | 131 | 139 | 200 | 204 | 287 | 291 | 190 | 206 | 190 | 190 |
| SR021.03 | 104 | 104 | 145 | 150 | 120 | 122 | 133 | 139 | 200 | 204 | 276 | 291 | 190 | 204 | 190 | 190 |
| SR021.38 | 104 | 106 | 145 | 161 | 120 | 120 | 133 | 133 | 196 | 204 | 263 | 291 | 172 | 172 | 190 | 190 |
| SR022.01 | 104 | 104 | 150 | 163 | 122 | 122 | 133 | 133 | 190 | 204 | 271 | 326 | 170 | 200 | 190 | 190 |
| SR022.03 | 104 | 104 | 142 | 150 | 122 | 122 | 125 | 133 | 190 | 203 | 271 | 326 | 172 | 198 | 190 | 190 |
| SR022.05 | 104 | 104 | 152 | 161 | 120 | 122 | 133 | 139 | 190 | 190 | 263 | 270 | 185 | 206 | 190 | 190 |
| SR022.07 | 104 | 106 | 157 | 157 | 118 | 120 | 125 | 133 | 169 | 182 | 267 | 271 | 172 | 185 | 190 | 190 |
| SR023.03 | 104 | 104 | 143 | 145 | 120 | 122 | 133 | 139 | 190 | 190 | 276 | 320 | 173 | 194 | 190 | 190 |
| SR023.31 | 106 | 106 | 143 | 145 | 122 | 133 | 133 | 133 | 176 | 186 | 292 | 334 | 210 | 212 | 190 | 190 |
| SR024.03 | 104 | 106 | 143 | 161 | 122 | 133 | 125 | 131 | 184 | 201 | 267 | 285 | 172 | 172 | 190 | 190 |
| SR024.08 | 104 | 104 | 157 | 163 | 122 | 132 | 133 | 133 | 186 | 198 | 270 | 321 | 198 | 200 | 190 | 190 |
| SR024.36 | 106 | 108 | 157 | 173 | 120 | 122 | 121 | 126 | 190 | 196 | 263 | 291 | 172 | 173 | 190 | 190 |
| SR025.01 | 104 | 106 | 152 | 159 | 122 | 122 | 125 | 133 | 193 | 226 | 287 | 291 | 170 | 177 | 190 | 190 |
| SR025.02 | 104 | 104 | 152 | 169 | 118 | 122 | 125 | 133 | 196 | 226 | 267 | 343 | 164 | 173 | 190 | 190 |
| SR025.07 | 106 | 106 | 139 | 159 | 118 | 118 | 133 | 133 | 200 | 226 | 326 | 328 | 172 | 172 | 190 | 190 |
| SR025.10 | 104 | 104 | 143 | 169 | 122 | 122 | 133 | 133 | 176 | 226 | 267 | 287 | 177 | 196 | 190 | 190 |
| SR026.05 | 104 | 106 | 142 | 159 | 120 | 122 | 126 | 133 | 200 | 237 | 266 | 267 | 173 | 175 | 190 | 190 |
| SR026.06 | 104 | 106 | 159 | 161 | 122 | 122 | 133 | 139 | 206 | 220 | 267 | 331 | 168 | 168 | 190 | 190 |
| SR026.53 | 104 | 106 | 139 | 165 | 118 | 122 | 133 | 133 | 192 | 196 | 266 | 289 | 183 | 200 | 190 | 190 |
| SR028.24 | 104 | 106 | 152 | 154 | 120 | 122 | 133 | 139 | 188 | 193 | 276 | 304 | 172 | 172 | 190 | 190 |
| SR028.32 | 104 | 104 | 143 | 157 | 118 | 120 | 133 | 139 | 188 | 190 | 263 | 326 | 172 | 175 | 190 | 190 |
| SR031.01 |  |  |  |  |  |  |  |  | 182 | 192 | 263 | 267 | 183 | 185 | 190 | 190 |
| SR031.02 | 104 | 106 | 135 | 145 | 120 | 122 | 125 | 133 | 182 | 192 | 263 | 270 | 183 | 185 | 190 | 190 |
| SR031.03 | 106 | 106 | 135 | 159 | 118 | 120 | 133 | 133 | 190 | 200 | 326 | 341 | 172 | 172 | 190 | 190 |
| SR032.02 | 163 | 169 | 130 | 142 | 120 | 120 | 123 | 131 | 226 | 230 | 273 | 273 | 172 | 172 | 190 | 190 |
| SR034.01 |  |  | 154 | 157 | 122 | 122 | 135 | 135 | 145 | 145 | 220 | 222 | 190 | 192 | 190 | 190 |
| SR034.02 |  |  | 157 | 157 | 122 | 122 | 135 | 135 | 145 | 145 | 210 | 212 | 170 | 192 | 190 | 190 |
| SR034.03 | 119 | 119 | 157 | 157 | 122 | 122 | 135 | 135 | 145 | 145 | 210 | 220 | 190 | 192 | 190 | 190 |
| SR039.01 | 171 | 179 | 130 | 165 | 120 | 120 | 131 | 135 | 200 | 200 | 204 | 273 | 158 | 158 | 192 | 192 |
| SR039.02 | 159 | 195 | 130 | 142 | 120 | 120 | 135 | 139 | 200 | 228 | 273 | 273 | 170 | 177 | 192 | 192 |
| SR040.01 | 163 | 165 | 139 | 139 | 116 | 116 | 117 | 123 | 200 | 208 | 173 | 287 | 158 | 170 | 192 | 192 |
| SR041.01 | 159 | 162 | 145 | 145 | 120 | 120 | 135 | 144 | 160 | 188 | 335 | 335 | 170 | 181 | 190 | 190 |
| SR041.03 | 104 | 104 | 154 | 161 | 120 | 120 | 133 | 137 | 160 | 220 | 202 | 335 | 179 | 181 | 190 | 192 |
| SR042.01 |  |  | 143 | 152 | 120 | 120 | 133 | 139 | 175 | 198 | 206 | 270 | 170 | 170 | 190 | 192 |
| SR042.02 | 104 | 104 | 143 | 152 | 120 | 120 | 135 | 137 | 162 | 198 | 177 | 270 | 173 | 181 | 190 | 190 |
| SR042.03 | 159 | 162 | 145 | 161 | 120 | 120 | 133 | 139 | 206 | 210 | 192 | 196 | 164 | 170 | 190 | 190 |
| SR043.01 | 154 | 155 | 130 | 130 | 120 | 120 | 126 | 135 | 175 | 200 | 200 | 271 | 170 | 172 | 192 | 192 |
| SR043.02 | 167 | 169 | 130 | 142 | 120 | 122 | 131 | 135 | 243 | 245 | 200 | 202 | 170 | 172 | 192 | 192 |
| SR044.02 | 148 | 163 | 130 | 130 | 120 | 120 | 131 | 135 | 224 | 228 | 273 | 273 | 170 | 170 | 190 | 190 |
| SR045.13 | 146 | 148 | 130 | 130 | 120 | 120 | 135 | 139 | 200 | 231 | 271 | 273 | 177 | 177 | 190 | 192 |
| SR047.08 | 104 | 106 | 143 | 150 | 122 | 122 | 133 | 133 | 198 | 204 | 263 | 324 | 173 | 173 | 190 | 190 |
| SR047.11 | 104 | 106 | 143 | 161 | 120 | 120 | 126 | 133 | 200 | 214 | 263 | 328 | 172 | 173 | 190 | 190 |
| SR047.17 | 104 | 106 | 130 | 157 | 120 | 122 | 126 | 133 | 190 | 190 | 263 | 328 | 168 | 179 | 190 | 190 |
| SR047.18 | 104 | 106 | 145 | 171 | 120 | 120 | 139 | 139 | 188 | 190 | 263 | 337 | 172 | 172 | 190 | 190 |
| SR048.01 | 104 | 106 | 150 | 159 | 120 | 125 | 133 | 133 | 169 | 188 | 263 | 266 | 156 | 173 | 188 | 188 |
| SR048.02 | 104 | 106 | 145 | 148 | 120 | 120 | 133 | 133 | 186 | 190 | 263 | 331 | 156 | 156 | 188 | 188 |
| SR048.06 | 108 | 115 | 150 | 159 | 120 | 132 | 133 | 133 |  |  | 266 | 266 |  |  |  |  |
| SR048.09 | 106 | 115 | 145 | 154 | 120 | 122 | 133 | 133 | 216 | 220 | 263 | 263 | 168 | 168 |  |  |
| SR052.01 | 104 | 115 | 157 | 157 | 120 | 132 | 139 | 139 | 186 | 190 | 263 | 263 | 156 | 156 | 188 | 188 |
| SR053.08 | 106 | 106 | 130 | 145 | 122 | 132 | 133 | 133 | 190 | 206 | 263 | 328 | 172 | 172 | 190 | 190 |
| SR054.01 |  |  |  |  |  |  |  |  | 186 | 200 | 263 | 263 | 156 | 156 | 188 | 188 |
| SR055.01 | 113 | 137 | 143 | 143 | 120 | 122 | 135 | 135 | 160 | 175 | 171 | 171 | 170 | 175 | 190 | 190 |
| SR055.02 | 140 | 142 | 167 | 167 | 116 | 120 | 135 | 144 | 175 | 188 | 171 | 192 | 170 | 224 | 190 | 190 |
| SR055.03 |  |  | 145 | 145 | 116 | 120 | 135 | 144 | 160 | 160 | 171 | 171 | 226 | 228 | 190 | 190 |
| SR055.04 | 104 | 104 | 143 | 143 | 116 | 120 | 135 | 144 | 160 | 160 | 171 | 331 | 170 | 170 | 190 | 190 |
| SR055.05 | 106 | 117 | 152 | 152 | 116 | 120 | 135 | 144 | 160 | 206 | 171 | 171 | 170 | 170 | 190 | 190 |
| SR058.01 | 130 | 130 | 169 | 171 | 135 | 137 | 139 | 139 | 201 | 206 | 263 | 263 | 168 | 168 | 190 | 190 |
| SR058.02 | 130 | 130 | 145 | 163 | 135 | 139 | 139 | 141 | 201 | 210 | 263 | 263 | 168 | 168 | 190 | 190 |
| SR059.01 | 102 | 106 | 128 | 148 | 120 | 120 | 133 | 139 | 196 | 203 | 308 | 313 | 170 | 177 | 190 | 190 |
| SR060.02 |  |  |  |  |  |  |  |  | 196 | 196 | 306 | 306 | 170 | 170 | 190 | 190 |
| SR060.05 | 106 | 106 | 150 | 150 | 120 | 120 | 133 | 139 | 196 | 196 | 306 | 306 | 170 | 170 | 190 | 190 |
| SR062.05 | 104 | 104 | 145 | 150 | 120 | 120 | 133 | 139 | 160 | 166 | 202 | 270 | 173 | 179 | 190 | 190 |
| SR062.06 |  |  |  |  | 120 | 120 | 133 | 137 | 190 | 208 | 171 | 196 | 170 | 181 | 190 | 190 |
| SR062.07 | 198 | 200 | 145 | 150 | 120 | 122 | 131 | 137 | 160 | 160 | 198 | 331 | 170 | 175 | 190 | 190 |
| SR062.08 | 104 | 104 | 145 | 145 | 120 | 120 | 133 | 137 | 160 | 190 | 194 | 270 | 170 | 179 | 190 | 190 |
| SR062.09 | 104 | 104 | 145 | 145 | 120 | 120 | 131 | 139 | 190 | 198 | 190 | 194 | 175 | 181 | 190 | 190 |
| SR064.04 | 159 | 196 | 137 | 139 | 122 | 122 | 117 | 123 | 233 | 235 | 208 | 210 | 170 | 172 | 190 | 190 |
| SR064.05 | 117 | 117 | 137 | 137 | 120 | 122 | 123 | 123 | 188 | 188 | 208 | 210 | 170 | 172 | 190 | 190 |
| SR066.11 | 115 | 115 | 152 | 159 | 120 | 120 | 117 | 123 | 226 | 235 | 285 | 291 | 173 | 173 | 190 | 190 |
| SR066.12 | 128 | 144 | 152 | 152 | 120 | 120 | 135 | 144 | 184 | 190 | 192 | 304 | 172 | 172 | 190 | 190 |
| SR066.13 | 119 | 128 | 145 | 171 | 120 | 120 | 126 | 141 | 180 | 198 | 292 | 334 | 172 | 188 | 190 | 190 |
| SR066.14 | 104 | 108 | 154 | 169 | 120 | 120 | 117 | 123 | 180 | 184 | 285 | 285 | 172 | 177 | 190 | 190 |
| SR067.08 | 106 | 128 | 148 | 148 | 120 | 120 | 119 | 126 | 186 | 201 | 194 | 196 | 172 | 172 | 190 | 190 |
| SR068.11 | 198 | 200 | 148 | 150 | 120 | 120 | 123 | 135 | 184 | 192 | 190 | 331 | 172 | 172 | 190 | 190 |
| SR068.13 | 115 | 115 | 148 | 150 | 120 | 120 | 126 | 126 | 184 | 190 | 326 | 331 | 172 | 172 | 190 | 190 |
| SR068.14 | 100 | 130 | 148 | 150 | 120 | 120 | 135 | 135 | 201 | 201 | 190 | 190 | 172 | 179 | 190 | 190 |
| SR068.15 | 115 | 134 | 148 | 148 | 120 | 120 | 126 | 139 | 184 | 192 | 198 | 266 | 172 | 172 | 190 | 190 |
| SR069.01 | 121 | 121 | 137 | 154 | 118 | 120 | 123 | 144 | 200 | 235 | 188 | 190 | 170 | 172 | 190 | 190 |
| SR070.05 | 121 | 121 | 135 | 139 | 120 | 120 | 135 | 135 | 210 | 210 | 181 | 186 | 172 | 172 | 190 | 190 |
| SR070.14 | 121 | 123 | 135 | 145 | 120 | 122 | 126 | 135 | 188 | 200 | 186 | 331 | 172 | 175 | 190 | 190 |
| SR072.01 | 104 | 104 | 161 | 161 | 122 | 122 | 141 | 141 | 200 | 200 | 192 | 200 | 170 | 172 | 190 | 190 |
| SR072.02 | 104 | 104 | 161 | 161 | 122 | 122 | 141 | 141 | 200 | 212 | 192 | 200 | 170 | 170 | 190 | 192 |
| SR073.01 | 188 | 190 | 133 | 152 | 124 | 124 | 137 | 139 | 192 | 212 | 171 | 196 | 172 | 177 | 190 | 190 |
| SR073.02 | 104 | 104 | 145 | 152 | 120 | 120 | 137 | 139 | 162 | 162 | 196 | 202 | 170 | 175 | 190 | 190 |
| SR073.03 | 104 | 104 | 152 | 161 | 118 | 118 | 139 | 139 | 160 | 160 | 202 | 204 | 170 | 173 | 190 | 192 |
| SR073.04 | 106 | 106 | 137 | 159 | 124 | 124 | 139 | 139 | 192 | 198 | 171 | 202 | 170 | 177 | 190 | 190 |
| SR073.05 | 104 | 104 | 161 | 161 | 120 | 120 | 139 | 139 | 160 | 198 | 202 | 204 | 170 | 170 | 190 | 192 |
| SR073.06 | 104 | 104 | 145 | 145 | 120 | 120 | 139 | 139 | 160 | 188 | 194 | 204 | 173 | 173 | 190 | 190 |
| SR074.02 | 206 | 208 | 130 | 142 | 120 | 120 | 135 | 139 | 200 | 200 | 271 | 323 | 170 | 177 | 190 | 190 |
| SR074.03 | 196 | 198 | 135 | 142 | 120 | 120 | 135 | 146 | 200 | 224 | 321 | 321 | 177 | 177 | 190 | 190 |
| SR074.04 |  |  |  |  |  |  |  |  | 224 | 226 | 271 | 297 | 172 | 172 | 190 | 192 |
| SR074.05 | 140 | 142 | 135 | 142 | 120 | 120 | 135 | 135 | 224 | 226 | 271 | 323 | 170 | 177 | 190 | 190 |
| SR074.07 | 140 | 140 | 135 | 142 | 120 | 120 | 135 | 146 | 200 | 200 | 271 | 323 | 177 | 177 | 190 | 190 |
| SR074.08 | 140 | 140 | 130 | 142 | 120 | 120 | 123 | 135 | 200 | 224 | 196 | 323 | 170 | 170 | 190 | 190 |
| SR074.09 | 198 | 200 | 130 | 135 | 120 | 120 | 123 | 135 | 200 | 200 | 196 | 323 | 170 | 177 | 190 | 190 |
| SR077.05 | 106 | 106 | 148 | 150 | 120 | 120 | 126 | 133 | 184 | 190 | 320 | 335 | 190 | 192 | 190 | 190 |
| SR077.06 | 142 | 142 | 145 | 145 | 122 | 122 | 133 | 146 | 188 | 193 | 282 | 294 | 160 | 187 | 190 | 192 |
| SR078.04 | 119 | 119 | 122 | 122 | 120 | 122 | 133 | 135 | 212 | 216 | 246 | 250 | 172 | 173 | 190 | 190 |
| SR078.06 | 119 | 121 | 145 | 148 | 120 | 122 | 135 | 135 | 200 | 216 | 240 | 242 | 156 | 172 | 188 | 188 |
| SR078.07 | 121 | 121 | 148 | 148 | 120 | 120 | 133 | 135 | 216 | 216 | 181 | 181 | 156 | 156 | 188 | 188 |
| SR078.08 | 121 | 121 | 122 | 122 | 120 | 122 | 133 | 133 | 200 | 216 | 240 | 242 | 156 | 156 | 188 | 188 |
| SR083.01 | 106 | 115 | 152 | 157 | 122 | 122 | 146 | 148 | 200 | 206 | 263 | 263 | 170 | 183 | 190 | 190 |
| SR088.01 | 123 | 134 | 139 | 139 | 120 | 120 | 133 | 133 | 198 | 198 | 192 | 194 | 156 | 156 | 188 | 188 |
| SR088.02 | 123 | 134 | 152 | 165 | 120 | 122 | 133 | 133 | 190 | 196 | 175 | 175 | 156 | 156 | 188 | 188 |
| SR089.01 | 142 | 142 | 148 | 148 | 120 | 120 | 133 | 133 | 184 | 192 | 171 | 171 | 156 | 156 | 188 | 188 |
| SR101.01 | 106 | 106 | 139 | 139 | 120 | 120 | 133 | 133 | 186 | 186 | 181 | 184 | 181 | 183 | 190 | 190 |
| SR101.02 | 106 | 106 | 139 | 139 | 120 | 120 | 133 | 144 | 186 | 186 | 270 | 335 |  |  | 190 | 190 |
